# Reduced circulating BMP10 and BMP9 and elevated endoglin are associated with disease severity, decompensation and pulmonary vascular syndromes in patients with cirrhosis

**DOI:** 10.1016/j.ebiom.2020.102794

**Published:** 2020-05-23

**Authors:** Nicola E. Owen, Graeme J. Alexander, Sambit Sen, Katherine Bunclark, Gary Polwarth, Joanna Pepke-Zaba, Anthony P. Davenport, Nicholas, W. Morrell, Paul. D. Upton

**Affiliations:** a Experimental Medicine and Immunotherapeutics (EMIT), University of Cambridge, Addenbrooke's Hospital, Cambridge, United Kingdom; b Institute for Liver and Digestive Health, University College London, Royal Free Hospital Pond St, Hampstead, London NW3 2QG, UK, Royal Free Hospital, London, United Kingdom; c Luton and Dunstable Hospital NHS Foundation Trust, Luton, United Kingdom; d Royal Papworth Hospital NHS Foundation Trust, Cambridge, United Kingdom; e Department of Medicine, University of Cambridge, Addenbrooke's Hospital, United Kingdom

**Keywords:** Bone morphogenetic protein 9, BMP10, Cirrhosis, Endoglin, ELISA

## Abstract

**Background:**

BMP9, originating from the liver, and BMP10 are circulating BMPs that preserve vascular endothelial integrity. We assessed BMP9, BMP10 and soluble endoglin (sEng) levels and their relationships to liver disease severity and associated pulmonary vascular syndromes in a cohort of well-characterised liver disease patients.

**Methods:**

Plasma samples from patients with liver disease (*n* = 83) and non-disease controls (*n* = 21) were assayed for BMP9, BMP10 and sEng. Levels were also assessed in a separate cohort of controls (*n* = 27) and PoPH patients (*n* = 8). Expression of mRNA and immunohistochemical staining was undertaken in liver biopsy specimens. Plasma BMP activity was assessed using an endothelial cell bioassay.

**Findings:**

Plasma BMP9 and BMP10 levels were normal in patients with compensated cirrhosis or fibrosis without cirrhosis, but markedly reduced in patients with decompensated cirrhosis, including those with hepatopulmonary syndrome (HPS) or portopulmonary hypertension (PoPH). Liver biopsy specimens revealed reduced mRNA expression and immunostaining for these ligands. Patient plasma samples with reduced BMP9 and BMP10 levels exhibited low BMP activity that was restored with exogenous BMP9. Endoglin mRNA expression was increased in cirrhotic livers and elevated circulating sEng levels in PoPH and HPS patients suggested increased endothelial sEng shedding in these syndromes.

**Interpretation:**

Plasma BMP9 and BMP10 levels are reduced in decompensated cirrhosis, leading to reduced circulating BMP activity on the vascular endothelium. The pulmonary complications of cirrhosis, PoPH and HPS, are associated with markedly reduced BMP9 and BMP10 and increased sEng levels, suggesting that supplementation with exogenous ligands might be a therapeutic approach for PoPH and HPS.

**Research in context**

**Evidence before this study**

BMP9 is a circulating vascular quiescence factor secreted by the liver. BMP10, a protein with close homology and similar endothelial activity to BMP9, is most highly expressed by the right atrium, with very low expression in the liver. Endoglin is a BMP9/10 co-receptor that can be shed from the cell surface as a soluble receptor (sEng). Imbalances of these proteins may associate with the vascular complications seen in severe cirrhosis, including hepatopulmonary syndrome (HPS) and portopulmonary hypertension (PoPH). Previous evidence has shown that BMP9 levels are reduced in cirrhosis and associate with the presence of portopulmonary hypertension.

**Added value of this study**

We found that plasma levels of BMP9 and BMP10 are reduced in patients with decompensated cirrhosis, whereas normal levels are present in patients with compensated cirrhosis or liver fibrosis short of cirrhosis, leading to reduced serum activity on endothelial cells. We also demonstrate reduced hepatic expression and synthesis of these proteins in cirrhotic livers. In addition, increased levels of sEng are found in patients with PoPH or HPS.

**Implications of all the available evidence**

The switch from compensated cirrhosis to decompensated disease in patients is associated with a dramatic reduction in life expectancy due to the development of complications associated with portal hypertension. We have identified that reduction of plasma BMP9 and BMP10 are strongly associated with decompensation. These reductions, in combination with raised sEng, are associated with PoPH or HPS. These proteins may constitute biomarkers that indicate the switch to decompensation and susceptibility to HPS and PoPH. Moreover, our findings suggest that supplementation with exogenous BMP9 or BMP10 might be a therapeutic strategy in decompensated cirrhosis and in PoPH and HPS, conditions with a major unmet need.

Alt-text: Unlabelled box

## 1 Introduction

Bone morphogenetic proteins (BMPs), belonging to the transforming growth factor-beta (TGF-β) superfamily, were first discovered to play important roles in bone and cartilage formation [1]. They are now known to regulate aspects of early development, organogenesis and cell differentiation, exerting a broader role as key regulators of mature tissue homeostasis. BMPs regulate cellular functions including proliferation, differentiation, migration and chemotaxis [1].

Bone morphogenetic protein 9 (BMP9), also known as growth differentiation factor 2 (GDF2), is expressed in murine liver by sinusoidal endothelial cells, hepatic stellate cells and Kupffer cells and can be detected in the circulation [2]. Like other BMPs, the inactive dimeric precursor protein (*ProBMP9*) is processed via furin in the liver to yield the N-terminal prodomain fragments and the active dimeric carboxy-terminal growth factor domain (BMP9 GFD), which remain in a non-covalent complex (Pro:BMP9) [2]. However, BMP9 circulates as a mixture of unprocessed *ProBMP9* and processed Pro:BMP9, the latter reported to comprise approximately 60% of the total plasma BMP9 pool [2]. In contrast, BMP10 is primarily expressed by the adult right atrium and is also secreted by mouse right atria as an active form in complex with its pro-domain (Pro:BMP10) [3], though we recently reported that the majority of plasma BMP10 is unprocessed *ProBMP10* [4]. A recent study reported that BMP10 is also expressed in the liver and that BMP9 and BMP10 might circulate as a biologically active heterodimer [5].

BMP9 and BMP10 are the most potent activators (EC50 approximately 50pg/ml) of vascular endothelial cell BMP signalling [6], [7], [8]. Typically, BMPs bind heterotetrameric membrane receptor complexes comprising two high affinity type I receptors (termed Activin like kinase receptors or ALKs) and two low affinity type II receptors (BMP receptor type II (BMPR-II), Activin A receptor type IIA (ACTR-IIA) or Activin A receptor type IIB (ACTR-IIB) [1]. Upon ligand binding, the constitutively active serine‑threonine kinase domain of the type II receptor phosphorylates and activates the type I receptor that in turn phosphorylates intracellular signalling molecules. Endothelial BMP9 and BMP10 signalling are mediated through complexes comprising the high affinity type I receptor, ALK1 and the type II receptors, BMPR-II and ACTR-IIA [6,7]. Functionally, BMP9 exerts a variety of protective effects on the adult vasculature, promoting endothelial quiescence, inhibiting apoptosis and maintaining barrier function [8,9]. Although less well characterised, BMP10 also induces high affinity endothelial ALK1 signalling and protects endothelial cells from apoptosis [3,6]. Endoglin, which is expressed in liver endothelial cells and hepatic stellate cells, is a homodimeric membrane glycoprotein that binds BMP9 and BMP10 with high affinity and regulates BMP signalling. Soluble endoglin (sEng) levels are reported to be elevated in liver fibrosis [10]. Both increased circulating and hepatic tissue levels of Endoglin appear to correlate with increased liver fibrosis stage [11].

Recent genetic data suggest an emerging role for loss of endothelial BMP9/10 signalling in pulmonary arterial hypertension (PAH). Inherited or *de novo* genetic mutations in the genes encoding BMP9 (*GDF2*), BMP10, ALK1 (*ACVRL1*) and endoglin (*ENG*) have been identified in some PAH cases [12], [13], [14]. Furthermore, heterozygous loss-of-function mutations of BMPRII account for 53–86% of familial PAH cases and 14–35% of idiopathic cases [15]. A role for a loss of BMP function in PAH is supported further by evidence that exogenous administration of BMP9 to rodent models of PAH improved haemodynamics, vascular remodelling and right ventricular hypertrophy [8].

Abnormalities of the pulmonary vasculature are well described in patients with advanced chronic liver disease, in particular the hepatopulmonary syndrome (HPS) and less often, portopulmonary hypertension (PoPH). HPS, found in up to 30% of patients with chronic liver disease [16,17], is characterised by the combination of intrapulmonary vascular dilatation and hypoxemia in those with chronic liver disease and/or portal hypertension. PoPH, defined as PAH in the presence of portal hypertension, is less common than HPS, affecting up to 8% of patients referred to liver transplant centres [18]. A recent study reported that plasma BMP9 levels are reduced in patients with PoPH and correlated to the severity of liver disease [19]. Conversely, a separate study reported increased serum BMP9 levels in patients with liver fibrosis [20], so the influence of liver disease on BMP9 regulation warrants further investigation. Furthermore, plasma sEng levels are also reported to be elevated in PoPH [19].

When we embarked on this study, data from studies with a synthetic dimeric sEng had suggested that sEng was an inhibitor of BMP9 signalling [19,21]. A recent study showed that endogenous sEng is monomeric and can bind to, but not inhibit BMP9 [22]. As sEng adenoviral overexpression in vivo can impair pulmonary vascular function via disruption of transforming growth factor-β (TGF-β) signalling [23] and changes in BMP9 and sEng were reported in PoPH [19], the potential additive consequences of changes in BMP9 and BMP10 along with sEng are important to consider. Therefore, the aim of the present study was to investigate the plasma levels of BMP9, BMP10 and sEng in a well-characterised cohort of patients with liver disease to assess whether the levels of these ligands change and if they segregate with disease severity or pulmonary vascular complications.

## 2 Materials and methods

### 2.1 Patients and plasma samples for ELISA

Plasma samples were collected (from 2014–2017) with informed consent from 83 (1–83) selected patients with liver disease and 21 (C1–C21) healthy controls with ethical approval obtained from the local research ethics committee (IRAS Project 83963). Demographic details are shown in eTable 1. Whole blood was collected in Monovette® EDTA tubes (Sarstedt, Leicester, Leicestershire, UK) chilled on ice and immediately centrifuged at 3000*xg* for 10 min at 4 °C. The plasma supernatant was transferred into polypropylene cryovials in 0.5 ml aliquots (maximum/vial) and stored at −80 °C. For this study, all patients with varying degrees of liver disease severity and aetiology were included between the ages of 18 and 78 years. For clinical assessment, 73 patients had a liver biopsy to confirm the stage of their liver disease within a year of plasma sampling. In the remaining 10 patients with cirrhosis where biopsy was not possible, disease severity was defined by clinical and radiological evidence of cirrhosis with portal hypertension.

Thirty-three (40%) of those patients with cirrhosis (Patients 51–83, eTable 1) underwent formal assessment for the presence of HPS or PoPH (including right heart catheterisation, bubble echocardiogram and direct portal venous pressure measurements), which were detected in 14 and 2 patients respectively according to agreed international criteria [24] (eTable 2 and eTable 3). Patients were recruited from among those patients having a liver transplant assessment and also from the cirrhosis clinic at Addenbrooke's Hospital, Cambridge. The study included patients with cirrhosis and evidence of portal hypertension via endoscopic or ultrasonographic assessment. Exclusion criteria were: age below 18 and above 70 years, known intrinsic cardiopulmonary disease, lacking the capacity to consent or pregnancy.

A further 8 samples from patients with confirmed PoPH (Po1-8, eTable 4) were obtained from Royal Papworth Hospital Research Tissue Bank with ethical approval from the research ethics committee (IRAS Project 247498) and a further 27 healthy controls (CP1-27, eTable 5) were collected (IRAS Project 83963).

Samples were assayed with ELISAs for BMP9, pBMP10 and sEng as described below, with randomised controls and patient samples evenly distributed across each assay plate. Operators were blinded to patient information until the data were analysed. Where sample volume was limiting, samples were assayed in the priority order of BMP9, BMP10 then sEng. Correlations were assessed with respect to three metrics of liver disease severity: United Kingdom Model for End-Stage Liver Disease (UKELD) [25], Model for End-Stage Liver Disease (MELD-Na) [26] and Child-Pugh Score (CPS) [27]. In addition, patients were analysed according to whether they exhibited compensated (no clinically overt ascites, no overt hepatic encephalopathy, no variceal haemorrhage and no jaundice) or decompensated (presenting any of the aforementioned symptoms) cirrhosis according to accepted definitions [28], [29], [30], [31].

### 2.2 Patients and biopsy samples for liver expression analyses

Human liver tissue was collected with informed consent under ethical approval obtained from the research ethics committee (IRAS Project 50805). Tissue was collected from 9 patients with end stage liver disease (all with decompensated cirrhosis) at the time of liver transplantation and 8 disease-control patients undergoing liver resection for colonic metastases with normal underlying liver confirmed histologically (eTable 6). Briefly, a sample approximately 2 cm^3^ was surgically removed, frozen immediately in liquid nitrogen and stored at −80 °C. Macroscopic analysis was undertaken to exclude the presence of HCC or other tumours in these samples and this was also monitored in the cut sections. Sections (10 µm) of frozen tissue were stained for BMP9 and BMP10 and examined using confocal microscopy. In addition, these samples were used to examine RNA expression of BMP9, BMP10 and endoglin. The patients from whom liver tissue was collected did not overlap with those sampled for plasma.

### 2.3 ELISAs for BMP9 and pBMP10

ELISAs were conducted using high binding 96-well ELISA plates (Greiner, Kremsmunster, Austria). For all incubation stages, plates were stored in a humidified chamber.

The BMP9 ELISA detects the free BMP9 GFD and Pro:BMP9, but not *ProBMP9* [4]. ELISA plates were coated with 0.5 µg/well of mouse monoclonal anti-human BMP9 antibody (MAB3209, R&D Systems, Abingdon, Oxon, UK) in PBS (10 mM phosphate pH 7.4, 137 mM NaCl, 2.7 mM KCl) overnight at 4oC. Plates were washed with PBS containing 0.05% (v/v) Tween-20 (PBS-T). Plates were then blocked with 1% (w/v) bovine serum albumin (BSA) in PBS (PBS/BSA) for 90 min at room temperature. Recombinant human BMP9 standards (4.88–5000 pg/ml) were diluted in PBS/BSA and 0.2% (v/v) goat serum (PBS/BSA/GS). Plasma samples (25 µl/well) were added to 75 µl/well PBS/BSA supplemented with 0.667% Triton x-100 and 0.266% GS. Samples and standards were incubated for 2 h at room temperature (RT). Plates were then washed with PBS-T followed by incubation with 0.4 µg/well biotinylated goat anti-human BMP9 antibody (BAF3209, R&D Systems) in PBS/BSA/GS for 2 h at room temperature. Assays were developed as described below. For analysis, BMP9 values that were interpolated as below the detection limit of 20pg/ml were assigned a value of 20 pg/ml.

The pBMP10 ELISA detects Pro:BMP10 and *ProBMP10*, but not the BMP10 GFD [4]. ELISA plates were coated with 0.5 µg/well of mouse monoclonal anti-human BMP10 antibody (MAB2926, R&D Systems) in PBS overnight at 4 °C. Plates were washed with PBS-T, then blocked with (PBS/BSA) for 90 min at room temperature. Purified recombinant human Pro:BMP10, expressed in HEK-EBNA cells, was kindly provided by Dr Wei Li (Department of Medicine, University of Cambridge, UK). Recombinant human Pro:BMP10 standards (97.65–100,000 pg/ml) were diluted in PBS/BSA/GS containing 4.5 mM EDTA. Plasma samples (30 µl/well) were added to 70 µl/well PBS/BSA supplemented with 0.714% Triton x-100, 0.286% GS and 6.42 mM EDTA. Samples and standards were incubated for 2 h at RT. Plates were then washed with PBS-T followed by incubation with 0.4 µg/well biotinylated goat anti-human BMP10 propeptide antibody (BAF3956, R&D Systems) in PBS/BSA/GS for 2 h at room temperature and developed as described below. For analysis, pBMP10 values that were interpolated as below the detection limit of 500 pg/ml were assigned a value of 500pg/ml.

To develop the BMP9 or pBMP10 ELISAs, plates were washed with PBS-T followed by incubation with ExtrAvidin®-Alkaline phosphatase (Sigma) diluted 1:400 in PBS/BSA for 90 min at RT. Plates were washed with PBS-T followed by water. The ELISA was developed in the dark at RT with a colourimetric substrate comprising 1 mg/ml 4-Nitrophenyl phosphate disodium salt hexahydrate (Sigma) in 1 M Diethanolamine, pH 9.8 containing 0.5 mM MgCl2 and the absorbance measured at 405 nm. Unknown values were extrapolated from the standard curve using a 4-parameter log curve fit.

### 2.4 ELISA for soluble endoglin

The soluble endoglin Quantikine assay was performed according to the manufacturer's instructions, plates were stored in a humidified chamber. Briefly, wells were coated with 100 µl monoclonal anti-human Eng antibody (360 µg/ml). After washing, wells were blocked with PBS/BSA, followed by incubation with sEng standard (15.63–8000pg/ml) diluted in PBS/BSA or plasma sample diluted 1:5 with PBS/BSA for 2 h at RT with shaking. Wells were washed three times with PBS-T and 100 µl human biotinylated anti-human endoglin conjugate (50 ng/ml) added to each well for 2 h at RT. Plates were washed three times, incubated with 100 µl streptavidin-HRP, washed again and then 100 µl substrate solution added. Plates were incubated for 30 min at RT in the dark and the development stopped by addition of 50 µl stop solution. The absorbance was read at 450 nm with baseline subtraction of the 550 nm values. Unknown values were extrapolated from the standard curve using a 4-parameter log curve fit.

### 2.5 Cell-based activity assay for BMP9 and BMP10

Human aortic endothelial cells were purchased from Lonza and maintained in EBM2 containing 5% FBS and supplied growth factors. For signalling assays, 2 × 10^5^ cells/well were seeded in 6-well plates and grown to confluence. Cells were then washed once with EBM2/0.1% (EBM2 containing 0.1% (v/v) FBS and Antibiotic-Antimycotic (A/A; 100 U/mL penicillin, 100 mg/mL streptomycin and 0.25 mg/mL amphotericin B, Invitrogen, Carlsbad, CA)). Cells were incubated overnight in EBM2/0.1%. For activity assays of control (*n* = 6) or liver disease patient (*n* = 16) plasmas, media were aspirated and 1.5 ml fresh EBM2/0.1% was added to each well and 15 µl of plasma or 100X BMP ligand added to the relevant wells. For spiking experiments, serial dilutions of Pro:BMP9 were prepared in control and patient plasmas. In all cases, cells were incubated for 1 h Total RNA was extracted using the RNeasy Mini Kit with DNAse digestion (Qiagen, West Sussex, UK). Samples were then analysed by QPCR for *ID1* and *ID2* as described below.

### 2.6 Quantitative PCR

cDNA was prepared from ~1 µg RNA (PAEC) or 400 ng RNA (liver tissue) using the High Capacity Reverse Transcriptase kit (Applied Biosystems, California, USA), according to the manufacturer's instructions. All qPCR reactions were prepared in a total volume of 10 µl using either 100 ng PAEC or 40 ng liver tissue cDNA with SYBR®Green Jumpstart™ Taq Readymix™ (Sigma-Aldrich, St Louis, MO), ROX reference dye (Invitrogen) and custom sense and anti-sense primers (200 nM each). Reactions were amplified on a QuantStudio 6 Flex 384-well PCR system (Applied Biosystems). Data were analysed using the comparative 2^−(∆∆Ct)^ method. For PAEC samples, the expression of each target gene was normalised to beta-2 microglobulin (*B2M*) and the difference in the amount of product produced was expressed as a fold change relative to the EBM2/0.1% control. For liver tissue samples, the arithmetic mean of the Ct values (Ct3HK) for *ACTB, B2M* and *HPRT* was calculated for each sample and relative expression determined as 2^−(CtGOI-Ct3HK)^. The primer sequences for *ACTB, B2M, BMP9, BMPR2, HPRT, ID1* and *ID2* are given in eTable 7 in full. *BMP10* and *ENG* expression was determined using QuantiTect primers from Qiagen.

### 2.7 ***Bmp9*^−/−^ mic**e

*Bmp9*^−/−^ mice (a kind gift from Professor Se-Jin Lee, Johns Hopkins University, Baltimore, USA) were bred with C57/Bl6J mice and heterozygotes back-crossed to generate *Bmp9*^−/−^ mice and wild-type littermates. Liver tissue was collected from 3 *Bmp9*^−/−^ and 3 wild type littermate mice. Sections (10 µm) of frozen tissue were stained for BMP9 and examined using confocal microscopy.

### 2.8 Haematoxylin and eosin staining

Fresh frozen human liver and portal vein tissue sections were thawed. Slides were stained with Mayer's Haematoxylin (Cat No: MHS80, Sigma Aldrich, Darmstadt, Germany) for 5 min followed by washing with distilled water. Scott's tap water (1.4 g sodium bicarbonate and 8 g magnesium sulphate dissolved in 400 ml of distilled water) was added to each slide for 1 min followed by washing with distilled water. Slides were counterstained with Eosin Y solution (Cat No: HT110280, Sigma Aldrich) for 5 min and washed with distilled water. Slides were then dehydrated serially for 1 min in 70% ethanol, twice in 100% ethanol, then xylene for 1 h and mounted with DPX mountant (Cat No: 44581, Sigma Aldrich).

### 2.9 Immunohistochemistry

Snap frozen tissue was used for immunohistochemistry. Sections (10 µm thickness) were cut from 8 control livers, 9 cirrhotic human livers, 3 wild-type mouse and 3 *Bmp9*^−/-^ mouse livers, apposed to positively-charged slides and air-dried. Slides were fixed by immersion in acetone at 4 °C for 10 min and then rehydrated in phosphate buffered saline (PBS) at room temperature. Slides were blocked for 2 h with PBS/5% Goat Serum (GS). Primary antibodies against BMP9 (AP2064A, 1:50 Abcepta, San Diego, CA), BMP10 (LS-C293026, 1:100, LSBio, Seattle, WA) and hepatocyte antibody (OCH1E5, 1:20, Thermo Fisher, Waltham, MA) were then added to human liver slides in PBS/Tween, 3% goat serum at 250 µl per slide. Primary antibodies for BMP9 (1:50) and BMP10 (1:100) were added to the mouse liver slides. The hepatocyte antibody was not used as it is mouse derived. Slides were then incubated overnight at 4 °C. Slides were then washed for 3 × 5 min with PBS/Tween followed by incubation with secondary antibodies goat anti-rabbit (GAR) Alexafluor 488 1:200, GAR Alexafluor 568 1:200 and Hoechst 1:100) in PBS/3% goat serum for 1 h. Slides were then washed for 3 × 5 min with PBS/Tween, mounted with Prolong Gold and images taken using Zeiss 510 Meta confocal microscope.

### 2.10 RNA extraction

Frozen human liver tissue samples (0.5 cm^3^), 8 control and 9 cirrhotic, were placed in lysing matrix D tubes (6913–050, MP Biomedicals, Escwege, Germany). TRIzol (1 ml) was added to samples homogenised using the MP FastPrep-24 Tissue Homogenizer for 45 s. This process was repeated until samples were homogeneous. RNA was then extracted using PureLink RNA Mini Kit (RNA Ambion, Life Technologies, Carlsbad, CA) according to the manufacturer's instructions. RNA quality and quantity were assessed using NanoDrop. Gene expression was then assessed by QPCR, as described in the methods supplement.

### 2.11 Statistical analysis

The D'Agostino and Pearson omnibus K2 test of normality was used to assess the distributions of the BMP9, pBMP10 and sEng ELISA data. All datasets were confirmed to not follow a normal distribution, so Mann Whitney or Kruskal–Wallis tests were used as appropriate. The Spearman coefficient was used for correlations. *P*-values <0.05 were considered statistically significant. Statistical analyses were performed using GraphPad Prism 8 for Windows (GraphPad Software, La Jolla, CA, USA). Further univariate and multivariate logistic regression analyses were undertaken, including interaction terms where appropriate, using R Studio v1.2.1335 [32] to assess the strength of association of plasma ligand levels with disease phenotypes and to examine potential ligand interactions.

## 3 Results

**Fig. 1:**
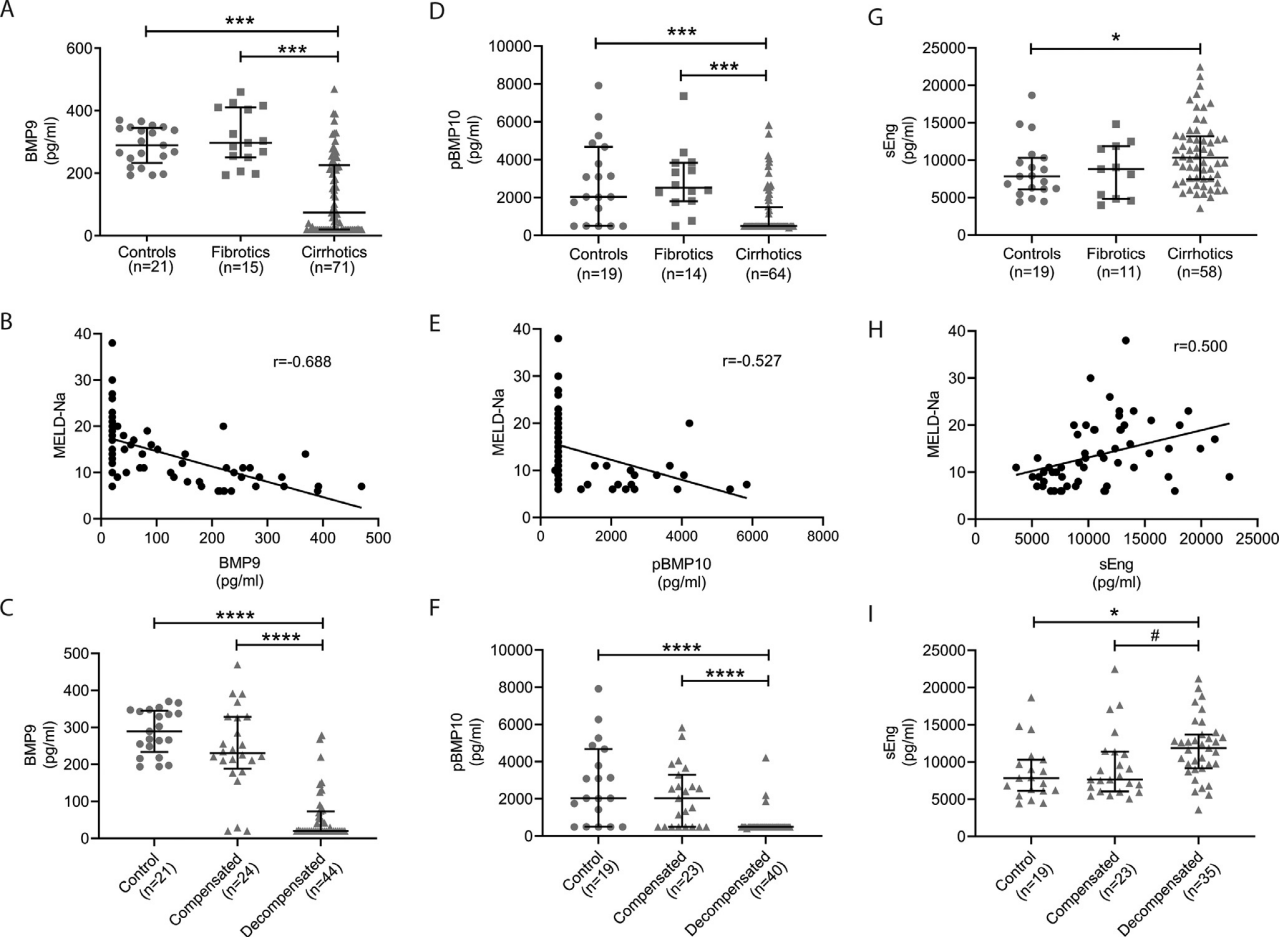
**Reduced plasma BMP9 and pBMP10 levels and increased sEng levels in cirrhosis are associated with decompensation.** (A,D,G) Plasma samples from controls or patients with either pre-cirrhotic liver fibrosis or with cirrhosis were assayed by ELISA for (A) BMP9, (D) pBMP10 or (G) sEng. Error bars show median and interquartile range. (B,E,H) Spearman correlation of: (B) BMP9 (*n* = 69, *P<*0.0001), (E) pBMP10 (*n* = 64, *P<*0.0001) or (H) sEng (*n* = 58, *P<*0.0001) levels with MELD-Na score of cirrhosis severity. (C,F,I) Patients were stratified for compensated and decompensated liver disease and plasma levels of (C) BMP9, (F) pBMP10 and (I) sEng compared between groups. Kruskal Wallis test: *P<0.05, ***P<0.001, ****P<0.0001, Mann Whitney Test #*P<*0.05.

Assay of plasma samples for circulating BMP9 (see eTable 8 for individual data) revealed that, compared to control subjects, a large proportion of patients with liver disease exhibited reduced levels of circulating BMP9 (Fig. 1A). This finding was restricted to patients with cirrhosis, rather than fibrosis (Fig. 1A). Increasing severity of liver disease as defined by either the MELD-Na, UKELD or Child-Pugh scores within the cirrhosis group correlated inversely with reduced circulating levels of BMP9 (Fig. 1B, eFig. 1A-B). Strikingly, plasma BMP9 levels did not differ between controls and patients with compensated liver disease, whereas BMP9 levels were significantly reduced in individuals with decompensated cirrhosis (Fig. 1C).

Similar to BMP9, the levels of circulating pBMP10 (see eTable 8 for individual data) were specifically reduced in patients with cirrhosis compared to patients with liver fibrosis or controls (Fig. 1D). Again, pBMP10 levels inversely correlated with increasing severity of liver disease indicated by the MELD-Na, UKELD or Child-Pugh scores (Fig. 1E, eFig. 1C–D) and we observed a striking reduction of plasma pBMP10 levels in decompensated patients compared to controls or patients with fibrosis or compensated liver disease (Fig. 1F). This was unexpected given the right atrium is considered the major source of BMP10, although low levels of BMP10 mRNA expression have been reported in liver [5]. Consistent with our recent observation in a cohort of PAH patients and controls [4], plasma BMP9 and pBMP10 levels correlated closely, suggesting some degree of co-regulation, though the measured levels of pBMP10 were ten times greater than those for BMP9 (eFig. 1E).

Endoglin is a cell-surface BMP9/BMP10 co-receptor that can be shed to release the soluble extracellular domain (sEng). The circulating levels of sEng (see eTable 8 for individual data) were higher in patients with cirrhosis compared to patients with fibrosis or controls (Fig. 1G). Patients with the most advanced cirrhosis had the highest levels of sEng (Fig. 1H, eFigure 2). Of note, plasma sEng levels were higher in decompensated patients when compared to controls or patients with compensated liver disease (Fig. 1I). The association between increased circulating levels of sEng in patients with cirrhosis confirm previous reports demonstrating that circulating levels of sEng correlate to fibrosis stage [11] or are elevated in cirrhosis [33]. Although previous studies have suggested reduced tissue BMP9 expression [34] and increased serum sEng levels in hepatocellular carcinoma patients [33,35], we did not observe any difference in levels of BMP9, pBMP10 and sEng between patients with cirrhosis and with HCC (eFigure 3).

Univariate logistic regression analysis of compensated versus decompensated cirrhotics indicated that BMP9 levels were significantly associated with decompensation (*P* = 0.031). Levels of pBMP10 and sEng were not significantly associated. Multivariate analysis did not predict a significant interaction between combinations of BMP9, pBMP10 and sEng.

**Fig. 2:**
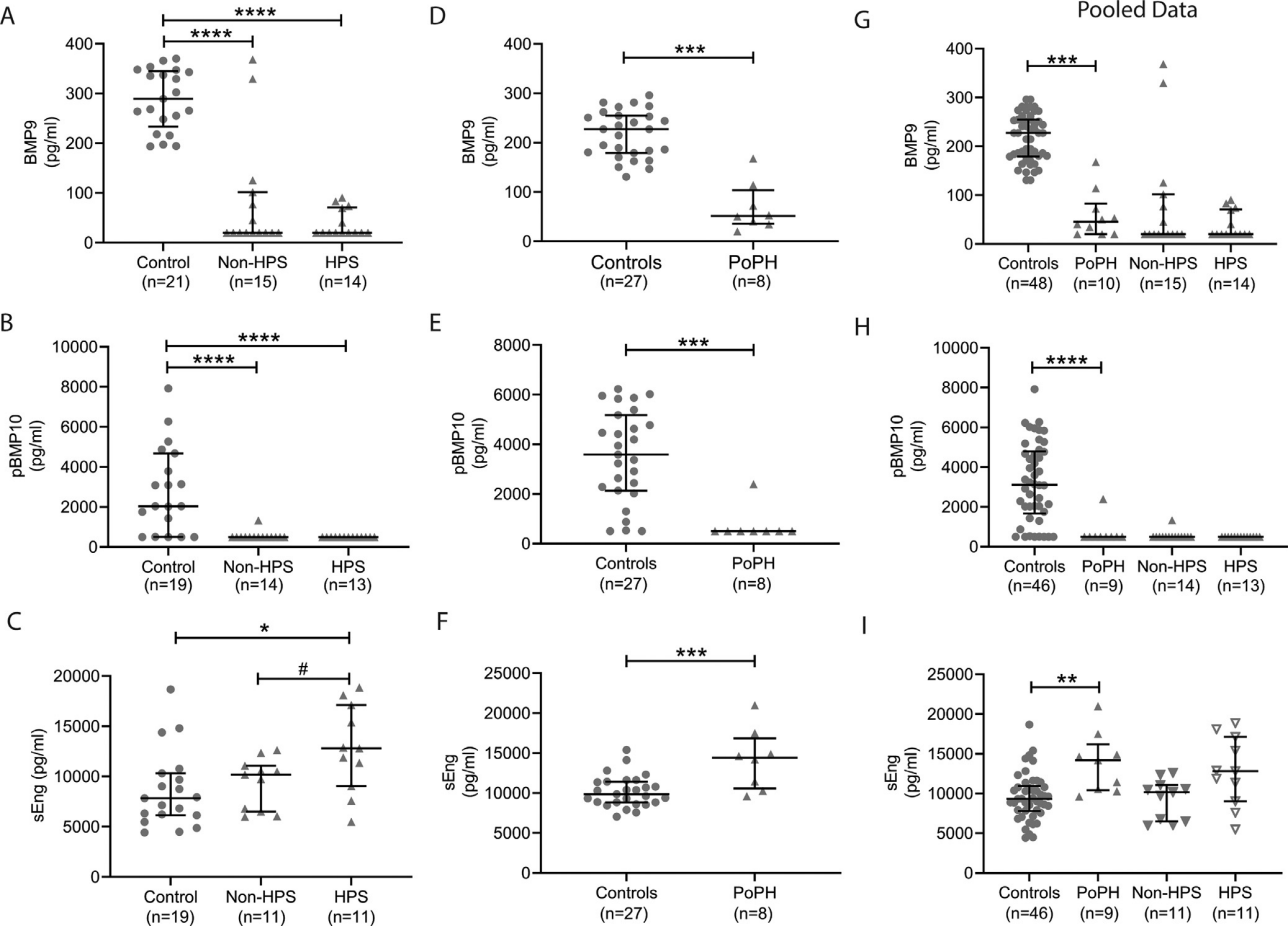
**Circulating BMP9 and pBMP10 levels are reduced in PoPH and HPS, whereas sEng levels are elevated in both syndromes.** Plasma samples ELISA data were for (A) BMP9, (B) pBMP10 or (C) sEng in cirrhotic patients were allocated according to the presence or absence of HPS, with controls shown for reference. Plasma samples from a separate cohort of controls and patients with confirmed PoPH were assayed by ELISA for (D) BMP9, (E) pBMP10 or (F) sEng. (G–I) The datasets for the control group samples and PoPH patients form both cohorts were combined and analysed to assess the levels of (G) BMP9, (H) pBMP10 and (I) sEng in the overall PoPH cohort. The data for the HPS patients are included in these graphs for reference. Error bars show median and interquartile range. Kruskal Wallis test: *P<0.05, **P<0.01, ***P<0.001, ****P<0.0001, Mann Whitney Test: #*P<*0.05.

**Fig. 3:**
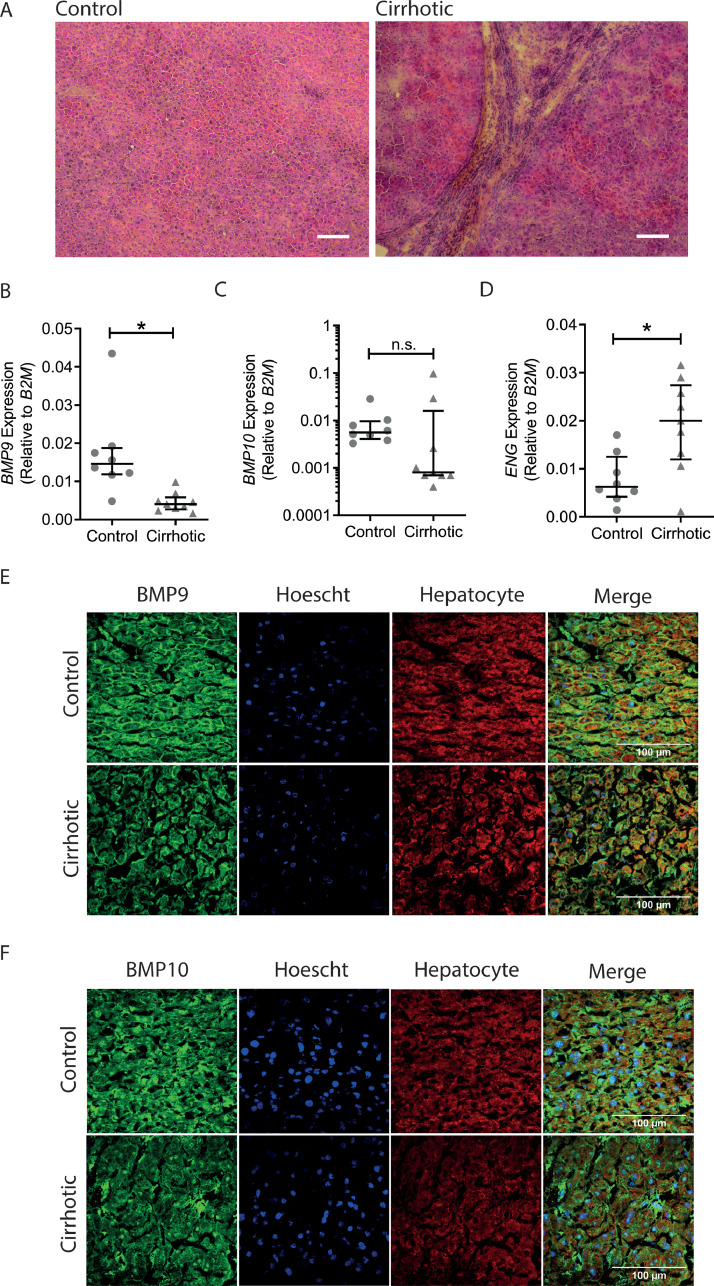
**Liver BMP9 and BMP10 are present in hepatocytes and their expression levels are reduced in cirrhosis, whereas endoglin expression is increased**. (A) Low power image (10X) of control and cirrhotic liver sections stained with haematoxylin and eosin (White scale bar length = 30 µm). (B–D) Total RNA was extracted from control (*n* = 8) and cirrhotic (*n* = 9) liver samples and analysed by qPCR for (B) *BMP9*, (C) *BMP10* and (D) *ENG* expression. Mann Whitney Test *P<0.05. (E,F) 8 healthy human liver samples and 9 samples from patients with cirrhosis were stained with Hoescht nuclear stain (blue) and a hepatocyte-specific antibody (red) in addition to antibodies (green) for (E) BMP9 or (F) BMP10. Images were captured using confocal microscopy and 100 µm scale bars are shown.(For interpretation of the references to color in this figure legend, the reader is referred to the web version of this article.)

The liver disease-associated pulmonary syndromes, HPS and PoPH, are both associated with increased mortality in liver disease patients [17,36,37]. Therefore, we further analysed those data for the 33 patients in whom formal assessment for the presence of HPS or PoPH had been undertaken (Patients 51–83, eTable 8). Compared to controls, patients with HPS on a background of cirrhosis and those without HPS had similar reductions in plasma levels of BMP9 (Fig. 2A) and pBMP10 (Fig. 2B). Also, no difference was observed between decompensated cirrhotics with or without HPS (eFig. 4A–B). Of note, sEng levels were higher in those cirrhosis patients with HPS compared to those without HPS (Fig. 3C). When the decompensated group were divided into HPS and non-HPS, only those patients with HPS exhibited a significant elevation of sEng compared to controls and compensated cirrhotics (eFigure 4C). The HPS cohort had significantly higher MELD-Na, UKELD and Child-Pugh Score when compared to the non-HPS patients with cirrhosis (eTable 9). Univariate logistic regression analysis of decompensated cirrhotics with HPS compared to those groups without HPS indicated that BMP9 levels exhibited an association with HPS (*Z* = 2.468, *P* = 0.014). Levels of pBMP10 and sEng were not significantly associated with HPS. Multivariate analysis did not predict a significant interaction between combinations of BMP9, pBMP10 and sEng.

In our cohort of patients, two were confirmed to have PoPH, both of whom had levels of BMP9 below the limit of detection, similar to all other patients with the most advanced liver disease. We obtained an additional 8 samples from patients with confirmed PoPH and a fresh cohort of control samples. We measured lower circulating levels of both BMP9 and pBMP10 and elevated sEng levels in the PoPH patients (Fig. 2D–F). We then undertook a combined analysis of the data for the controls and PoPH patients from both cohorts, again showing that levels of BMP9 and pBMP10 are significantly reduced in PoPH, whilst sEng levels were increased (Fig. 2G–I, HPS data included on these figures for comparison). Univariate logistic regression analysis for the association of BMP9, pBMP10 and sEng with PoPH in the single cohort of 27 controls and 8 PoPH patients indicated that BMP9 (*P* = 0.036) and BMP10 levels (*P* = 0.0075) were significantly associated with PoPH, whereas sEng levels were not associated. When the data for the controls and PoPH patients from both cohorts were combined, the predictive power of BMP9 increased (*P* = 0.0275), pBMP10 slightly decreased (*P* = 0.011) and sEng showed a significant association (*P* = 0.025). Multivariate analysis to examine the effect of BMP9, pBMP10 and sEng in combination did not predict a significant interaction for either the single cohort or combined datasets.

**Fig. 4:**
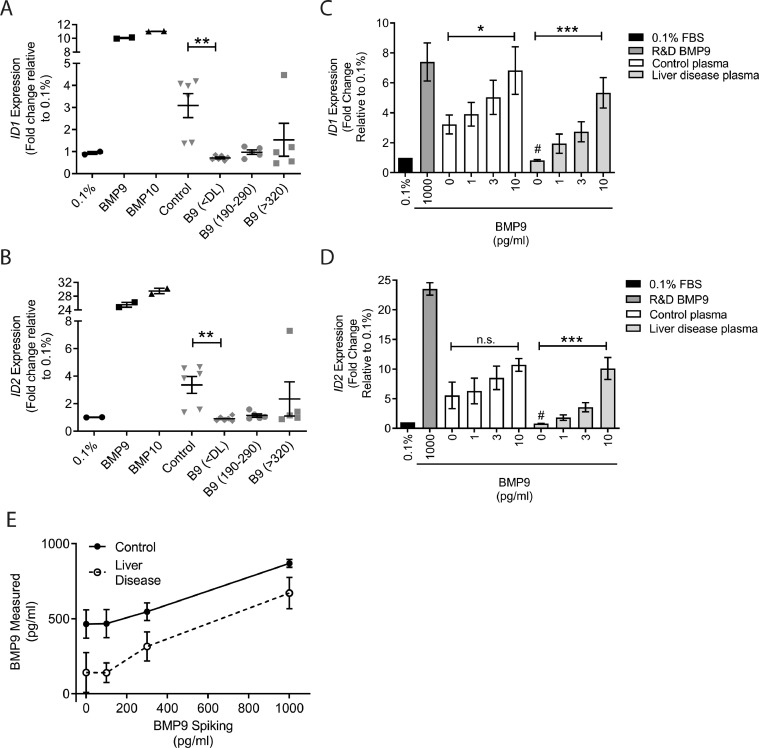
**BMP activity is low in liver disease but can be reconstituted with exogenous BMP9.** (A,B) HAECs were serum-depleted overnight followed by addition of 1 ng/ml BMP9, BMP10, or 1% plasma from controls (*n* = 6) or liver disease patients with undetectable (<DL; *n* = 6), normal (190–290; *n* = 5) or elevated (>320; *n* = 5) levels of BMP9. After 1 h, cells were lysed, RNA extracted and cDNA analysed for the expression of (A) *ID1* or (B) *ID2*. (C and D) Plasmas (1% final concentration) from controls (*n* = 4) or liver disease patients (*n* = 4) were added alone, or after spiking with ProBMP9, to serum depleted HAECs for 1 h. After 1 h, cells were lysed, RNA extracted, and cDNA analysed for the expression of (C) *ID1* or (D) *ID2*. (E) The same spiked plasma samples (*n* = 4 control and 4 liver disease) as in (C) and (D) were assayed using the BMP9 ELISA. *P<0.05, **P<0.01, ***P<0.001 Kruskal Wallis test, ^#^*P<*0.05 Mann–Whitney test compared to control plasma.

To determine whether the markedly reduced levels of BMP9 found in advanced cirrhosis was explained by reduced hepatic synthesis of BMP9, further studies were undertaken on liver tissue obtained from patients with cirrhosis at the time of liver transplantation compared with healthy human liver tissue (Fig. 3A). BMP9 mRNA was detected in both healthy and cirrhotic liver, but expression was significantly reduced in cirrhotic liver (Fig. 3B). BMP10 mRNA expression was also tended to be reduced, but the reduction in mRNA levels was not significant (Fig. 3C). Endoglin mRNA expression was increased in cirrhotic liver samples compared to controls (Fig. 3D). In parallel, sections of liver tissue were stained for BMP9 (Fig. 3E) and BMP10 (Fig. 3F). BMP9 protein was predominantly localised to the cell boundaries of hepatocytes in healthy human liver with reduced expression in cirrhotic samples (Fig. 3E), consistent with synthetic failure underlying reduced plasma levels in cirrhosis. The specificity of the antibody to BMP9 was confirmed by immunohistochemical staining of wild type mice and a murine *Bmp9* knock-out (eFigure 5A). In human liver, BMP10 staining was also prevalent in hepatocytes, though the distribution appeared more diffuse than the localisation of BMP9 (Fig. 4F). Again, BMP10 staining was reduced in cirrhotic livers (Fig. 4F). As a control to show that the antibody was specifically staining BMP10, we demonstrated high levels of staining in human right atrium and low staining in human left ventricle (eFigure 5B), consistent with known the levels of BMP10 mRNA expression in these tissues [3].

BMP9 and BMP10 are the major BMPs mediating plasma and serum-derived BMP signalling activity in endothelial cells, as shown by several neutralisation studies [[2], [3], [4],^9^,^19^]. This circulating BMP9/10 activity is considered to be a key mechanism to preserve vascular function in the adult [9]. We therefore assessed endothelial BMP9/BMP10 activity, represented by 1 h *ID1* and *ID2* transcriptional responses. The *ID1* and *ID2* responses were reduced in plasma from cirrhosis patients compared to healthy controls (Fig. 4A-B). Intriguingly, even those samples from cirrhotic patients with normal measured BMP9 levels demonstrated reduced activity. One explanation might be the presence of a factor antagonising the BMP ligands or downstream signalling. To address this, exogenous Pro:BMP9 was added at concentrations of 100, 300 and 1000 pg/ml to plasma samples from healthy controls or patients with liver disease. Plasma samples from control patients stimulated robust basal *ID1* and *ID2* responses (Figs. 4C-D), which were enhanced slightly by exogenous Pro:BMP9. The addition of Pro:BMP9 to plasma samples from patients with liver disease reconstituted the *ID1* and *ID2* responses in a dose dependent manner (Figs. 4C-D), with concentration-response curves similar to those with BMP9 in normal media (eFigure 6), suggesting that the reduced activity was not due to antagonism by another factor. Exogenous Pro:BMP9 was detected in a concentration-dependent manner in both control and liver disease patient plasma (Fig. 4E).

## 4 Discussion

In this study, we report that plasma levels of both BMP9 and pBMP10 are markedly reduced in patients with cirrhosis, but not in patients with pre-cirrhotic liver fibrosis. Among the cirrhotic patients, BMP9 and pBMP10 levels were normal in compensated individuals, but were dramatically reduced in those with decompensated liver disease. This was accompanied by increased plasma sEng levels in decompensated individuals. Moreover, BMP9 and pBMP10 levels were reduced in patients with hepatopulmonary syndrome or PoPH and sEng was elevated. Ex vivo analysis of patient plasma demonstrated that cirrhosis leads to reduced plasma BMP activity on endothelial cells that can be recovered through spiking with exogenous BMP9. The latter confirms that low BMP9 and pBMP10 levels lead to reduced activity, rather than the presence of factors that are inhibiting BMP signalling.

BMP9 is known to be predominantly derived from the liver [2]. We suggest that the reduction of BMP9 levels we observe is due to reduced hepatic expression, as indicated from our mRNA expression data. These data are consistent with a recent analysis of a publicly available microarray dataset from control and cirrhotic livers, though the authors acknowledged that they did not observe differences in three other datasets [34]. In mice, chronic hepatic injury induced by carbon tetrachloride or diethoxicarbonyl-1,4-dihydrocollidine results in reduced BMP9 expression [19,38,39]. Furthermore, acute injury induced either by partial hepatectomy, a single carbon tetrachloride dose or lipopolysaccharide exposure results in a transient reduction of liver BMP9 expression [38]. In general, these studies indicate that BMP9 expression is reduced as a result of physical damage and/or inflammatory stimuli, consistent with the processes underlying the pathology of cirrhotic liver disease.

In this study, we show in human liver sections that BMP9 and BMP10 proteins are localised to the borders of hepatocytes and this is reduced in cirrhotic liver. This suggests that human hepatocytes may secrete these ligands, although like other TGF/BMP ligands, it is possible that BMP9/BMP10 may be associated with extracellular matrix proteins [38]. Our observation of hepatocyte BMP9 staining is consistent with a previous report of BMP9 immunostaining in hepatocytes in human and mouse liver [2]. Furthermore, these authors examined a human liver cell cDNA panel in which highest BMP9 expression was identified in intrahepatic biliary epithelial cells, followed by hepatocytes (15% expression compared to the biliary epithelial cells), with low expression in hepatic stellate cells and hepatic sinusoidal endothelial cells (both <1% compared to the biliary epithelial cells) [2]. This contrasts with other reports in rodents that suggest that BMP9 is predominantly expressed by hepatic stellate cells [5,38,40]. This raises two possibilities, either the cellular expression of BMP9 and BMP10 differ between rodents and humans, or these ligands are highly expressed by hepatic stellate cells, but then sequester in the extracellular matrix. In cirrhotic livers, the tissue architecture was disrupted, with more intercellular spaces, so the reduction in BMP9 staining could be due to a lack of expression or reduced matrix sequestration. As we also demonstrate reduced BMP9 mRNA expression in the cirrhotic livers, our data suggest reduced synthesis is more likely. This is consistent with the reduction of *Bmp9* expression in mice where the liver architecture has been disrupted by partial hepatectomy [38].

Our observation of reduced plasma BMP9 associating with liver disease is consistent with two recent studies of PoPH patients [19,41], though comparisons of compensated versus decompensated patients have not been reported. Conversely, our data showing no change of plasma BMP9 in fibrotic patients contrasts with the increased serum BMP9 levels reported in fibrotic patients, especially those with stage 4 fibrosis [20]. In our study, we assayed BMP9 levels in plasma rather than serum and optimised the ELISA protocol to mitigate against false positive or negative findings that commonly arise in ELISAs due to plasma matrix interference and heterophilic antibody crosslinking [42]. The latter could be significant since hyperimmunoglobulinaema is prevalent in patients with liver disease and increases with severity [43]. Similar to Nikolic et al. we include goat serum to mitigate heterophilic antibody crosslinking [19]. We also include Triton X-100 to reduce matrix interference and improve recovery [4].

Our observation that pBMP10 was reduced in the plasma of cirrhotic patients was intriguing, because BMP10 mRNA is most highly expressed by the right atrium in the adult [3]. Consistent with a recent in situ hybridisation study [5], we show by immunohistochemistry and PCR that both BMP9 and BMP10 are expressed in the liver, although BMP10 expression levels are low. Active BMP10 is expressed and secreted by the mouse right atrium as a prodomain-associated complex, though the proportion of Pro:BMP10 and *ProBMP10* secreted is not known [3]. Moreover, BMP10 signalling constitutes the endothelial cell BMP responses via ALK1 in zebrafish [44,45], so cardiac-derived BMP10 contributes to the active circulating pool. Therefore, the reduction of plasma pBMP10 may be due to reduced hepatic expression or the diseased liver causing reduced pBMP10 release from the right atrium. From our recent study, it seems that the majority of BMP10 in peripheral plasma is unprocessed *ProBMP10*, suggesting that this may be activated by furin-type proteases on specific destination cells [4]. This was supported by our observation that the BMP10 GFD ELISA, which cross-reacts with Pro:BMP10 and the BMP10 GFD, but not *ProBMP10*, only detects very low levels of GFD in plasma [4]. During the preparation of this manuscript, Rochon et al. published data demonstrating a reduction of the BMP10 GFD in PoPH patients, but not those with HPS [41]. Again, the levels of the BMP10 GFD were very low and similar to our calculated limit of detection for the GFD ELISA, suggesting that measuring BMP10 GFD in plasma is challenging [45]. This contrasts with the high levels detected by the pBMP10 ELISA, which has equal cross-reactivity for Pro:BMP10 and ProBMP10, but does not detect the BMP10 GFD [4]. Consistent with low plasma BMP10 GFD levels in humans, Tillet et al. reported that the mouse BMP10 GFD was present at approximately 70pg/ml compared to BMP9 levels of 500 pg/ml [5], consistent with the recent data in man [45]. These low levels of BMP10 GFD suggest that active Pro:BMP10 from the right atrium may be taken up by the vascular endothelium proximal to the right atrium, as suggested in zebrafish studies [44]. Although we detect higher levels of inactive unprocessed ProBMP10 in plasma than the measured levels of BMP9, previous studies using immunoneutralisation indicated that the majority of plasma or serum-derived endothelial BMP activity is due to BMP9 [[2], [3], [4],^9^,^19^].

The highly significant correlation between plasma BMP9 and pBMP10 levels is consistent with our recent study in controls and PAH patients and suggests some degree of co-regulation [4]. Recently, Tillet et al. reported that BMP9 and BMP10 are present in the circulation as heterodimers [5]. However, our data in this study and from 120 control individuals previously [4] indicate that pBMP10 levels in control subjects are 10-fold higher than BMP9 levels. Also, the BMP10 GFD is measured at lower levels than BMP9 [5,41] This suggests that these two ligands are not circulating at a 1:1 stoichiometry and thus heterodimers are unlikely to account for the entire circulating pool [4].

We observed reduced plasma BMP signalling activity on endothelial cells in patients with liver disease, even when normal plasma levels of BMP9 and pBMP10 were measured. As inflammation is prevalent in liver disease [46], this led us to question whether the plasma samples may contain inflammatory factors that interfere with BMP signalling. However, spiking exogenous BMP9 reconstituted the plasma activity of cirrhotic plasma, implying that repression of signalling by inhibitory factors is unlikely to explain the reduced activity. The damaged liver also produces reactive oxygen species and BMP9 is susceptible to oxidation, so this could possible explain the normal levels but reduced activity in some patients [47].

In cirrhosis, we identified that plasma BMP9 and BMP10 were reduced and sEng was increased, with the measured levels correlating to disease severity scores. Subdivision of the cirrhotic cohort into compensated and decompensated patients revealed that decompensation is associated with a dramatic reduction of plasma BMP9 and pBMP10 and increased sEng levels. This switch is clinically important as compensated disease has a median survival of >12 years, whereas decompensation associated with the development of ascites results in death after approximately 2 years [28]. Reduced BMP9 and pBMP10 and elevated sEng are likely to be a manifestation of a dramatic reduction of liver function and could directly lead to the onset of the clinical complications associated with decompensation. As these proteins are key regulators of vascular function, their dysregulation may underlie the changes that occur in portal hypertension and the associated pulmonary haemodynamic complications. We found that BMP9 and pBMP10 levels were reduced in patients with PoPH, but were similar to those in other patients with advanced cirrhosis. It is possible that the reduction in BMP9 and BMP10 might favour evolution to PoPH, as suggested previously [19]. There may also be subclinical pulmonary hypertension, where pulmonary artery (PA) pressures are elevated but are insufficient to reach diagnostic criteria. We may be missing these patients as PA pressures are not measured routinely in all of those with cirrhosis. As we only had 2 confirmed PoPH cases in the cohort of 33 cirrhosis patients assessed for pulmonary haemodynamics, we measured BMP9 and pBMP10 levels in a second cohort of patients with PoPH. Consistent with the recent study by Nikolic et al., we observed reduced plasma BMP9 in PoPH and an increase in sEng [19]. In addition, we showed that pBMP10 levels were dramatically reduced in patients with PoPH. This is intriguing as we also identified reduced BMP9 and pBMP10 in cirrhosis, regardless of the presence or absence of HPS. In contrast, higher levels of plasma sEng were observed HPS in compared to those without HPS and although not significant, sEng levels were higher in decompensated patients with HPS compared to decompensated patients without HPS. Therefore, it is possible that reduced BMP9 and pBMP10 levels represent a marker of decompensation in advanced liver disease, increasing the susceptibility to PoPH and HPS. During the preparation of this manuscript, a letter reported reductions of plasma BMP9 in patients with HPS or PoPH compared to liver disease patients without these syndromes, whereas BMP10 levels were only reduced in HPS [41]. However, this study did not address the potential association with decompensation, which we have found to be a delineating factor.

Elevated sEng may represent a biomarker of vascular injury or the progressive increase of systemic inflammation in decompensation [31] and further increases may associate with the onset of PoPH and HPS. Certainly, the association with PoPH is consistent with the reported association of increased plasma soluble endoglin with pulmonary hypertension [19]. Furthermore, in pre-eclampsia, levels of sEng, proposed to be derived from the placenta, are elevated and thought to contribute to the pulmonary hypertensive state [23]. Although we show that ENG mRNA expression is increased in cirrhotic livers compared to controls, we cannot say if this alone corresponds to increased sEng shedding. It is known that endothelial cells can release sEng via cleavage of cell surface endoglin by MT1-MMP [48], but sEng shedding by other cell types has not been assessed. MMPs can be regulated by several mechanisms, including differential expression, cellular localisation or regulation of proteolytic activity [49]. A limitation of our study in terms of exploring this mechanism further is that we are restricted by the availability of fresh biopsy samples for comparison of sEng production by livers of PoPH/HPS patients with non-PoPH/HPS cirrhotic patients.

Mechanistically, it has been proposed, from studies using commercially sourced sEng-Fc, which is a dimer, that sEng is an inhibitor of BMP9 signalling [19,21]. However, a recent study demonstrated that endogenous sEng is monomeric and although it can displace the pro-domain to bind the BMP9 GFD, it does not inhibit BMP9 signalling [22]. The mechanism by which sEng may influence the pulmonary vasculature is not entirely clear. In vitro, dimeric recombinant sEng inhibits TGFβ-mediated NOS-dependent vasodilatation [23] and inhibits pulmonary endothelial cell network formation in Matrigel assays [50], but whether endogenous monomeric sEng has the same effect is not known. In these studies, adenoviral overexpression of sEng in vivo promoted increases in mean arterial pressure in adult rats [23] and impaired neonatal rat lung vascularisation [50]. In light of this, we hypothesise that reduced BMP9/10 production leading to reduced endothelial function, when combined with the additional impact of sEng on other factors, such as TGFβ, at the hepatic or pulmonary level, may promote HPS or PoPH.

This study is consistent with the identification of BMP9 mutations in PAH patients [12], [13], [14]. These mutations lead to reductions of plasma BMP9 and pBMP10 levels and BMP activity in patients harbouring pathogenic mutations associated with PAH [4]. Furthermore, some patients with PAH who did not harbour BMP9 mutations also exhibited reduced BMP9 and pBMP10 levels that were associated with reduced plasma activity with no evidence of liver disease [4]. A limitation of this study is that our cohort comprises small numbers of patients with HPS or PoPH with differing severities, so we cannot extrapolate any information relating to these biomarkers and severity of pulmonary syndromes. However, our data provide a rationale for studying of larger cohorts to assess whether these biomarkers associate with severity and assessment of longitudinal samples for monitoring disease decompensation and transition to pulmonary syndromes.

In conclusion, we show that advanced liver disease leads to reductions in the expression of BMP9 and BMP10 in the liver leading to reduced plasma levels and activity on endothelial cells. It is possible that loss of pulmonary endothelial homeostasis due to low circulating levels of BMP9 and BMP10 in combination with increased sEng plays a pathobiological role in HPS and PoPH. If so, supplementation with exogenous ligands might provide a therapeutic approach for these major pulmonary vascular complications of cirrhosis.

## Declaration of Competing Interest

PDU is a founder of, and scientific advisor to Morphogen-IX Ltd. NWM is a founder and CEO of Morphogen-IX Ltd. PDU and NWM have US (US10336800) and EU (EP3166628B1) patents entitled: “Therapeutic Use of Bone Morphogenetic Proteins.” All other authors declare no competing interests.

## Appendix Supplementary materials

Image, application 1
