# Supplementary material for: Reduced circulating BMP10 and BMP9 and elevated endoglin are associated with disease severity, decompensation and pulmonary vascular syndromes in patients with cirrhosis
Source: eBioMedicine. 2020 May 23;56:102794. doi: 10.1016/j.ebiom.2020.102794 (PMC7248419; doi:10.1016/j.ebiom.2020.102794)
Supplement: Supplementary file 1 [file mmc1.pdf]

Reduced circulating BMP10 and BMP9 and elevated endoglin are associated with disease severity, decompensation and pulmonary vascular syndromes in patients with cirrhosis

Nicola E. Owen MBBS [1], Graeme J. Alexander MD FRCP [2], Sambit Sen MD FRCP [3], Katherine Bunclark [4], Gary Polwarth MSc [4], Joanna Pepke-Zaba PhD [4], Anthony P. Davenport ScD [1], Nicholas, W. Morrell ScD [5#], Paul. D. Upton PhD [5#]

[1]Experimental Medicine and Immunotherapeutics (EMIT), University of Cambridge, Addenbrooke's Hospital, Cambridge, UK

[2] Institute for Liver and Digestive Health, University College London, Royal Free Hospital Pond St, Hampstead, London NW3 2QG, UK , Royal Free Hospital, London, United Kingdom.

[3] Luton and Dunstable Hospital NHS Foundation Trust, Luton, UK

[4] Royal Papworth Hospital NHS Foundation Trust, Cambridge, UK

[5] Department of Medicine, University of Cambridge, Addenbrooke's Hospital, UK

[#] joint senior authors

<u>SUPPLEMENTARY MATERIALS</u>

Expanded Methods

eTable 1: Demographics and values of biomarkers for severity calculations for cirrhotic and fibrotic patients assessed in this study.

eTable 2: Summary of diagnostic criteria and staging in HPS and PoPH.

eTable 3: Clinical and haemodynamic measurements for the 33 patients who underwent right heart catheterisation and bubble echocardiogram.

eTable 4: PoPH patient demographics and measured levels of BMP9, pBMP10 and sEng.

eTable 5: Demographics of control patients for PoPH patient plasma ELISAs and measured levels of BMP9, pBMP10 and sEng.

eTable 6: Demographics of patients and controls sampled for liver biopsies analysed by QPCR and immunohistochemistry**.**

eTable 7: Sequences of oligonucleotide primers used for QPCR.

eTable 8: Comorbidities, liver disease severity scores and measured values for BMP9, pBMP10 and sEng for individual cirrhotic and fibrotic patients and non-disease controls assessed in this study.

eTable 9: Liver disease severity scores for cirrhotic patients classified as non-HPS and HPS.

eFigure 1: BMP9 and pBMP10 levels correlate to the severity of liver disease and to each other.

eFigure 2: sEng levels correlate to the severity of liver disease.

eFigure 3. Circulating BMP9, pBMP10 and sEng do not significantly differ between patients with or without hepatocellular carcinoma.

eFigure 4: Circulating BMP9 and pBMP10 are reduced to similar extends in decompensated cirrhotics with or without HPS and sEng levels are elevated in both groups.

eFigure 5. Validation of the BMP9 and BMP10 antibodies used for immunohistochemistry.

eFigure 6: BMP9 stimulates *ID* gene transcription to a maximum at 100pg/ml in HAECs.

ID	Age	Sex	Aetiology	Fibrosis Stage	Blood results							Asc	HE
					Bilirubin (µmol/l)	ALB (g/l)	Na (mmol/l)	Cr (µmol/l)	Plt (10*9/l)	PT (sec)	INR		
1	33	F	HCV/ArLD	Cirrhosis	75	28	131	90	49	19.7	1.7	3	2
2	58	F	ArLD/A1AT	Cirrhosis	72	33	134	90	107	23.4	2	3	2
3	54	M	HCV	Cirrhosis	622	23	132	174	43	30.8	2.7	3	2
4	48	M	AIH	Mild	19	43	139	80	192	12.6	1.1		
5	58	M	AIH	Mild	10	39	139	77	188	11.6	1		
6	45	F	AIH	Mild	12	40	138	61	258	11.1	1		
7	54	F	AIH/ A1AT	Cirrhosis	22	40	137	70	166	10.8	1	1	0
8	46	M	HCV	Mild	4	40	145	86	275	11.4	1		
9	45	M	AIH	Severe	12	37	140	62	122	12.8	1.1		
10	42	F	PBC/AIH	Cirrhosis	16	34	139	62	133	12	1	1	0
11	53	M	HCV/ArLD	Cirrhosis	17	23	144	52	123	14.5	1.3	1	0
12	60	M	HCV	Cirrhosis	10	31	141	40	59	11	1	1	0
13	42	F	ArLD	Cirrhosis	15	33	134	66	215	16.9	1.5	2	0
14	57	M	HCV	Cirrhosis	10	44	140	63	190	12.3	1.1	1	0
15	78	F	PSC	Moderate	9	39	139	63	236	10	0.9		
16	72	F	AIH	Moderate	9	40	142	54	230	12.5	1.1		
17	63	M	ArLD	Cirrhosis	61	30	138	76	35	19.7	1.7	2	2
18	71	M	AIH	Mild	26	46	140	78	261	12.6	1.1		
19	55	F	PSC	Moderate	10	44	141	73	217	12.6	1.1		
20	67	F	PBC/AIH	Cirrhosis	19	35	142	52	164	10.8	1	1	0
21	43	F	AIH	Mild	7	38	138	61	226	12	1		
22	46	F	PBC/AIH	Cirrhosis	37	33	138	60	180	10.5	1	1	0
23	46	F	AIH	Mild	6	35	139	57	283	11	1		
24	53	F	AIH	Cirrhosis	8	39	138	70	211	12.6	1.1	1	1
25	72	F	AIH	Cirrhosis	44	28	136	62	110	15.1	1.4	2	0
26	47	M	HCV	Cirrhosis	10	44	138	54	108	11.6	1	1	0
27	72	F	AIH	Cirrhosis	10	37	140	71	139	11.3	1	1	0
28	73	M	AIH	Moderate	6	37	138	73	149	12.6	1.1		
29	22	F	AIH	Cirrhosis	5	38	139	41	192	11.3	1	1	0
30	39	F	AIH	Cirrhosis	9	37	139	63	114	11.1	1	1	0
31	46	M	ArLD	Cirrhosis	13	42	139	70	103	11.7	1		0
32	53	M	ArLD	Cirrhosis	20	30	138	100	168	14.7	1.3	1	0
33	46	M	HCV	Cirrhosis	10	44	141	73	176	11.4	1	1	0
34	59	M	HCV	Cirrhosis	5	44	135	61	296	10.8	0.9	1	0
35	50	F	ArLD	Cirrhosis	15	46	139	71	133	12.2	1.1	1	0
36	60	F	HCV	Cirrhosis	20	46	142	55	80	12.4	1.1	1	0
37	63	M	HCV	Cirrhosis	16	38	139	55	125	13.1	1.2	1	0
38	69	F	ArLD/ HCC	Cirrhotic	144	24	137	39	203	19.8	1.6	1	0
39	85	M	HCC	Mild	17	42	141	75	154	12.8	1.1		
40	67	F	NAFLD/ HCC	Cirrhosis	258	17	136	77	173	16.5	1.4	1	1
41	60	M	HBV/ HCC	Cirrhosis	9	39	140	79	109	15.7	1.4	1	0
42	66	M	ArLD/ HCC	Cirrhosis	28	32	137	86	79	13.2	1.1	2	0
43	35	M	HBV/ HCC	Cirrhosis	24	37	138	71	34	17.2	1.5	1	0
44	73	M	NAFLD/ HCC	Cirrhosis	24	26	131	149	91	14.2	1.2	2	1
45	70	M	ArLD/ HCC	Cirrhosis	49	29	134	67	127	15.6	1.3	3	0
46	68	M	ArLD/ HCC	Cirrhosis	9	41	141	102	133	12.8	1.1	1	0
47	71	M	NALFD/ArLD/HCC	Cirrhosis	13	30	143	52	44	13.2	1.1	1	0
48	72	M	NAFLD/HCC	Severe	11	41	138	98	270	11.6	1		
49	46	F	ArLD/HCC	Cirrhosis	93	26	133	30	95	16.3	1.4	1	0
50	64	M	NAFLD/ HCC	Cirrhosis	14	41	140	92	165	16.4	1.4	2	0
**51**	**69**	**F**	**PBC**	**Cirrhosis**	**29**	**28**	**139**	**46**	**140**	**12.6**	**1.2**	**2**	**0**
**52**	**57**	**M**	**HCV**	**Cirrhosis**	**25**	**28**	**135**	**21**	**59**	**15.1**	**1.3**	**2**	**0**
**53**	**44**	**M**	**HCV/ArLD/ mild HPS**	**Cirrhosis**	**78**	**24**	**139**	**114**	**139**	**18.5**	**1.6**	**2**	**0**
**54**	**60**	**M**	**NAFLD/ArLD**	**Cirrhosis**	**91**	**31**	**132**	**108**	**64**	**19.9**	**1.6**	**2**	**0**
**55**	**48**	**M**	**ArLD/mod HPS**	**Cirrhosis**	**153**	**33**	**131**	**70**	**138**	**25.9**	**2.2**	**3**	**2**
**56**	**57**	**F**	**AIH/PBC/mod HPS**	**Cirrhosis**	**74**	**27**	**139**	**62**	**68**	**14.3**	**1.2**	**1**	**2**
**57**	**59**	**F**	**NASH**	**Cirrhosis**	**13**	**34**	**126**	**97**	**55**	**12**	**1**	**3**	**1**
**58**	**65**	**M**	**ArLD**	**Cirrhosis**	**18**	**29**	**136**	**103**	**86**	**15.1**	**1.3**	**3**	**0**
**59**	**46**	**M**	**HCV/ArLD**	**Cirrhosis**	**13**	**27**	**142**	**61**	**121**	**14.1**	**1.4**	**3**	**0**
**60**	**66**	**M**	**HBV/NAFLD**	**Cirrhosis**	**35**	**27**	**135**	**79**	**111**	**13.8**	**1.4**	**1**	**0**
**61**	**46**	**M**	**ArLD/mod HPS**	**Cirrhosis**	**29**	**25**	**135**	**78**	**71**	**18.7**	**1.6**	**3**	**0**
**62**	**53**	**F**	**ArLD/mild HPS**	**Cirrhosis**	**56**	**23**	**133**	**78**	**106**	**17**	**1.5**	**2**	**0**
**63**	**54**	**M**	**HCV/PoPH**	**Cirrhosis**	**38**	**25**	**130**	**87**	**54**	**14.6**	**1.3**	**1**	**0**
**64**	**60**	**F**	**Sarcoid**	**Cirrhosis**	**20**	**30**	**135**	**83**	**96**	**12**	**1.1**	**3**	**0**
**65**	**63**	**M**	**PSC**	**Cirrhosis**	**21**	**26**	**141**	**111**	**41**	**12.5**	**1.1**	**3**	**0**
**66**	**66**	**M**	**NAFLD/mod HPS**	**Cirrhosis**	**88**	**26**	**136**	**87**	**90**	**18.1**	**1.6**	**3**	**0**
**67**	**66**	**F**	**HCV**	**Cirrhosis**	**17**	**34**	**140**	**76**	**85**	**12.5**	**1.1**	**1**	**0**
**68**	**63**	**F**	**NAFLD**	**Cirrhosis**	**18**	**34**	**132**	**78**	**93**	**13.1**	**1.2**	**1**	**0**
**69**	**62**	**M**	**ArLD/severe HPS**	**Cirrhosis**	**31**	**35**	**135**	**123**	**122**	**15**	**1.3**	**3**	**0**
**70**	**58**	**M**	**ArLD/PoPH**	**Cirrhosis**	**108**	**35**	**138**	**107**	**103**	**15**	**1.3**	**1**	**0**
**71**	**40**	**F**	**ArLD/mod HPS**	**Cirrhosis**	**185**	**30**	**137**	**57**	**46**	**23.2**	**2**	**1**	**0**

72	52	M	ArLD	Cirrhosis	11	31	135	98	148	13.9	1.2	3	0
73	54	M	HCV/mod HPS	Cirrhosis	54	26	134	89	42	16.9	1.4	2	0
74	66	M	ArLD	Cirrhosis	27	31	131	103	75	17	1.5	3	1
75	58	M	ArLD/mod HPS	Cirrhosis	29	23	139	117	120	22.4	1.9	3	0
76	49	M	Sarcoid/v severe HPS	Cirrhosis	12	33	132	107	113	11.2	1	1	0
77	60	M	NAFLD	Cirrhosis	54	28	139	75	145	16.7	1.4	1	0
78	64	F	ArLD	Cirrhosis	30	32	134	65	79	17.1	1.3	3	0
79	59	M	NAFLD	Cirrhosis	68	26	137	87	176	18.1	1.4	2	1
80	58	M	ArLD/mod HPS	Cirrhosis	100	29	134	125	87	24.1	2	3	0
81	55	F	ArLD	Cirrhosis	129	27	124	48	87	25	2.2	3	0
82	51	M	A1AT/v severe HPS	Cirrhosis	27	37	137	60	57	17.1	1.5	1	0
83	66	F	HCV/mild HPS	Cirrhosis	18	25	138	58	59	16.6	1.5	1	1

eTable 1: Demographics and values of biomarkers for severity calculations for cirrhotic and fibrotic patients assessed in this study.

The demographics for all patients (n=83) assessed for fibrosis/cirrhosis are detailed. Of these, patients 51-83 were assessed for cardiopulmonary parameters by right heart catheterisation, portal venous pressure measurements and bubble echocardiogram. Those patients highlighted in blue have hepatopulmonary syndrome (HPS, n=14) with severity indicated for individual patients (mod = moderate, v = very). Those patients in red have PoPH (n=2). **Abbreviations: A1AT** - alpha 1 anti-trypsin, **AIH** - autoimmune hepatitis, **ALB** – albumin**, ArLD** - alcohol related liver disease, **Asc** – ascites, **Cr** – creatine, **HBV** - hepatitis B virus, **HCC** - hepatocellular carcinoma, **HCV** - hepatitis C virus, **INR** - international normalised ratio, **Na** - sodium, **NAFLD** - non-alcoholic liver disease, **PBC** - primary biliary cholangitis, **Plt** – platelets, **PSC** - primary sclerosing cholangitis, **PT** - prothrombin time. **Scoring systems: Ascites**: Child-Pugh Score for ascites - 1: none, 2: mild (or suppressed with medication), 3: moderate to severe (or refractory), **Hepatic encephalopathy (HE)** using West Haven criteria - 0: none, 1: changes in behaviour with minimal change in level of consciousness, 2: gross disorientation, drowsiness, inappropriate behaviour, 3: marked confusion, incoherent speech, sleeping but rousable to vocal stimuli, 4: comatose. **Grey boxes:** no data or sample.

Diagnostic criteria	HPS	PoPH
	Portal hypertension, with or without liver disease<br><br>Intrapulmonary shunting confirmed by positive contrast-enhanced echocardiography (or >6% uptake in the brain with radionucleotide lung perfusion scanning)<br><br>$PA\text{-}aO_2$ ≥15 mmHg (2 kPa) or PaO_2 <80 mmHg (10.7 kPa)<br><br>Patients > 64 years: $PA\text{-}aO_2$ ≥20 mmHg (2.7 kPa) or a PaO_2 <70 mmHg (9.3 kPa)	Portal hypertension, with or without liver disease<br><br>Mean pulmonary arterial pressure (MPAP) >25 mmHg at rest<br><br>Mean pulmonary artery occlusion pressure (MPAOP) <15 mmHg<br><br>Pulmonary vascular resistance (PVR)* >240 dyn/sec/cm^5 or 3 Wood units (mmHg/litre/min)†
**Stage** PaO_2 in mmHg (kPa), MPAP in mmHg	**Mild:** PaO_2 ≥80 (10.7)<br><br>**Moderate:** PaO_2 ≥60 to <80 (≥8–<10.7)<br><br>**Severe:** PaO_2 ≥50 to <60 (≥6.7–<8)<br><br>**Very severe:** <50 (6.7) or <300 (40) on 100% oxygen	**Mild:** MPAP > 25 to < 35<br><br>**Moderate**: MPAP ≥ 35 to < 45<br><br>**Severe**: MPAP ≥ 45

eTable 2: Summary of diagnostic criteria and staging in HPS and PoPH. *PVR (dyn/sec/cm^5) = 80 x (MPAP–MPAOP)/cardiac output (in litre/min). †Some previous studies have used a PVR cut-off of 120 dyn/sec/cm^5. **MPAP** = mean pulmonary arterial pressure; **PaO_2** = partial pressure of arterial oxygen; **$PA\text{-}aO_2$** = alveolar-arterial oxygen gradient. Adapted from Sen et al [1]

ID	Aetiology	Fibrosis stage	PaO_2 (mmHg)	A-a gradient ($PA-aO_2$)	HVPG (mmHg)	mPAP (mmHg)	PAWP (mmHg)	PVR (Wood Units)	CO (L/min)
51	PBC	Cirrhosis	12.4	1.62	11	18	14	1.1	3.7
52	HCV	Cirrhosis	15.1	0.57	30	7	4	0.5	5.94
53	HCV/ArLD/ mild HPS	Cirrhosis	12.3	2.58	18	10	9	0.1	8.11
54	NAFLD/ArLD	Cirrhosis	11.3	3.70	8	11	6	0.5	9.97
55	ArLD/mod HPS	Cirrhosis	8.8	5.70	8	17	11	0.9	6.71
56	AIH/PBC/mod HPS	Cirrhosis	10.3	3.39	13	21	15	0.8	7.74
57	NASH	Cirrhosis	10.9	3.48	17	12	8	0.8	5.27
58	ArLD	Cirrhosis	11.9	2.35	9	15	12	0.7	4.48
59	HCV/ArLD	Cirrhosis	10.1	3.40	16	16	9	1.5	4.77
60	HBV/NAFLD	Cirrhosis	7.0	4.88	14	27	16	2.8	3.92
61	ArLD/mod HPS	Cirrhosis	8.9	5.48	11	26	21	0.7	7.05
62	ArLD/mild HPS	Cirrhosis	12.7	2.43	17	11	9	0.4	5.47
63	HCV/PoPH	Cirrhosis	13.4	0.98	24	66	10	15.6	3.58
64	Sarcoid	Cirrhosis	11.5	3.25	12	12	9	0.9	3.36
65	PSC	Cirrhosis	15.0	0.50	27	13	7	0.8	7.8
66	NAFLD/mod HPS	Cirrhosis	8.8	6.33	9	20	13	0.4	16
67	HCV	Cirrhosis	12.5	1.87	9	18	12	2.0	3.05
68	NAFLD	Cirrhosis	10.1	2.53	18	31	14	2.5	6.8
69	ArLD/severe HPS	Cirrhosis	7.7	4.94	5	47	37	2.5	4.02
70	ArLD/PoPH	Cirrhosis	10.4	3.85	15	47	3	14.8	2.98
71	ArLD/mod HPS	Cirrhosis	9.1	4.53	27	23	20	0.8	3.89
72	ArLD	Cirrhosis	11.7	3.05	25	29	18	1.6	6.98
73	HCV/mod HPS	Cirrhosis	10.5	3.77	28	19	13	0.7	8.61
74	ArLD	Cirrhosis	12.1	2.40	22	10	5	0.7	7.22
75	ArLD/mod HPS	Cirrhosis	10.5	2.50	17	24	18	1.0	6.16
76	Sarcoid/v severe HPS	Cirrhosis	6.0	9.25	12	12	9	0.4	8.34
77	NAFLD	Cirrhosis	12.9	0.85	39	19	15	0.6	7.05
78	ArLD	Cirrhosis	13.2	1.42	9	41	31	2.4	4.25
79	NAFLD	Cirrhosis	9.8	4.33	29	14	9	0.6	8.51
80	ArLD/mod HPS	Cirrhosis	8.3	6.08	19	25	22	0.3	8.95
81	ArLD	Cirrhosis	8.8	5.60	20	30	31	0.1	5.43
82	A1AT/v severe HPS	Cirrhosis	6.3	7.83	9	20	12	1.1	9.8
83	HCV/mild HPS	Cirrhosis	11.6	3.15	18	19	17	0.6	7

eTable 3: Clinical and haemodynamic measurements for the 33 patients who underwent right heart catheterisation and bubble echocardiogram. Those patients highlighted in blue have hepatopulmoanry syndrome (HPS, n=14) with severity indicated for individual patients (mod = moderate, v = very), those in red have PoPH. Abbreviations: **A-a gradient** – alveolar-arterial oxygen gradient, **CO** – cardiac output, **HVPG** – hepatic venous pressure gradient, **mPAP** - mean pulmonary artery pressure, **PAWP** - pulmonary artery wedge pressure, **PaO_2** – partial pressure of arterial oxygen, **PVR** - pulmonary vascular resistance.

Patient	Po1	Po2	Po3	Po4	Po5	Po6	Po7	Po8
Age	51	57	51	70	61	45	47	44
Sex	F	F	M	F	M	M	M	F
Aetiology	ArLD	ArLD/ paracetamol	HCV/ArLD	NASH	HCV/ArLD	ArLD	ArLD	ArLD
Stage	Cirrhosis	Cirrhosis	Cirrhosis	Cirrhosis	Cirrhosis	Cirrhosis	Cirrhosis	Cirrhosis
Bilirubin	51	34	13	26	33	20	24	59
Albumin	28	42	33	34	33	43	32	39
Na	139	132	138	138	137	143	140	143
Creatinine	76	80	64	161	61	88	86	57
PT	16.5	14.5	NA	12.3	12.7	11.1	14.5	13.7
INR	1.4	1.2	1.1	1.0	1.1	1.1	1.0	1.2
BMP9 (pg/ml)	50.2	167.9	72.2	34.4	53.0	40.5	114.3	20.0
pBMP10 (pg/ml)	500	2397	500	500	500	500	500	500
sEng (pg/ml)	20997	14202	14882	17515	14633	10280	11445	9606
Ascites	1	1	1	1	1	1	1	1
HE	0	0	0	0	0	0	1	0
WHO Functional Class	3	2	3	2	3	3	3	3
Exercise Capacity (6MWD)	350	333	423	297	309	371	217	343
mPAP (mmHg)	39	51	57	39	51	68	41	57
PAWP (mmHg)	4	8	11	3	8	12	11	8
PVR (Wood Units)	9	10.5	9.8	10.9	11	10.8	6.7	13.2
Cardiac output (L/min)	3.9	4.1	4.7	3.3	3.9	5.2	4.5	3.7

eTable 4: PoPH patient demographics and measured levels of BMP9, pBMP10 and sEng.
The demographics for the PoPH patients (n=8) are provided. **Abbreviations: HCV** - hepatitis C virus, **ArLD** - alcohol related liver disease, **NASH** - non-alcoholic steatohepatitis, **Na** - sodium, **PT** - prothrombin time, **INR** - international normalised ratio, **mPAP** - mean pulmonary artery pressure, **PAWP** - pulmonary artery wedge pressure, B - pulmonary vascular resistance. **Scoring systems: Ascites**: Child-Pugh Score for ascites - 1: none, 2: mild (or suppressed with medication), 3: moderate to severe (or refractory), **Hepatic encephalopathy (HE)** using West Haven criteria - 0: none, 1: changes in behaviour with minimal change in level of consciousness, 2: gross disorientation, drowsiness, inappropriate behaviour, 3: marked confusion, incoherent speech, sleeping but rousable to vocal stimuli, 4: comatose

Sample ID	Sex	Age	BMP9 (pg/ml)	BMP10 (pg/ml)	sEng (pg/ml)
CP1	M	47	183.7	2284	11634
CP2	M	36	147.0	527	7566
CP3	M	58	194.7	4469	8454
CP4	F	32	130.9	*500*	14140
CP5	M	38	150.4	875	11626
CP6	F	21	262.2	6231	9642
CP7	F	44	163.9	*500*	10704
CP8	F	41	234.9	3376	8554
CP9	M	40	189.5	3234	9341
CP10	F	21	244.1	2636	12325
CP11	F	51	272.4	5875	8847
CP12	F	24	227.4	3953	8885
CP13	F	24	296.1	5835	9399
CP14	M	34	215.0	4629	9860
CP15	M	46	170.6	2021	8852
CP16	M	53	180.4	1300	10301
CP17	M	60	179.1	2136	9313
CP18	F	54	255.0	5392	8608
CP19	F	56	281.4	6020	12829
CP20	F	46	241.0	4196	10389
CP21	F	47	253.2	4416	11420
CP22	F	63	281.4	5954	11356
CP23	M	65	162.6	2917	7045
CP24	M	62	227.4	2443	7908
CP25	F	68	274.2	5185	10801
CP26	M	38	250.8	4775	15405
CP27	M	60	186.3	3593	10414

eTable 5: Demographics of control patients for PoPH patient plasma ELISAs and measured levels of BMP9, pBMP10 and sEng.

The demographics for the controls (n=27) assayed for comparison to the PoPH patient samples are provided.

ID	Age	Sex	Aetiology	Fibrosis Stage	Blood results							Varices	Asc	HE
					Bilirubin (µmol/l)	Alb (g/l)	Na (mmol/l)	Cr (µmol/l)	Plt ($x10^9/l$)	PT (sec)	INR			
DT1	42	M	ArLD	Cirrhosis	20	35	128	130	193	14.5	1.2	1	3	0
DT2	48	F	AIH/PSC	Cirrhosis	112	22	138	93	103	25.2	2.1	0	2	0
DT3	54	M	NAFLD	Cirrhosis	29	24	137	94	54	18.2	1.7	1	3	1
DT4	52	M	HCV	Cirrhosis	41	30	141	88	57	17.0	1.5	1	1	0
DT5	64	M	ArLD	Cirrhosis	48	28	135	50	52	15.8	1.4	2	3	0
DT6	48	F	HCV	Cirrhosis	14	31	137	60	53	15.6	1.2	2	3	0
DT7	48	M	AIH	Cirrhosis	48	24	136	45	29	17.1	1.4	3	3	0
DT8	58	M	HBV	Cirrhosis	16	31	137	98	60	11.9	1.1	3	1	1
DT9	64	M	ArLD	Cirrhosis	135	21	136	70	101	22.3	1.9	1	2	0
CT1	56	M	Metastatic sigmoid colon cancer	None										
CT2	61	F	Metastatic colon cancer	None										
CT3	76	M	Metastatic rectal cancer	None										
CT4	78	M	Metastatic rectal cancer	None										
CT5	45	F	Metastatic choroidal melanoma	None										
CT6	47	F	Metastatic colon cancer	None										
CT7	74	F	Metastatic gastric GIST	None										
CT8	74	F	Metastatic colon cancer	None										

eTable 6: Demographics of patients and controls sampled for liver biopsies analysed by QPCR and immunohistochemistry. Abbreviations: **AIH** - autoimmune hepatitis, **Alb** – albumin, **ArLD** - alcohol related liver disease, **Asc** – ascites, **Cr** – creatine, **HBV** - hepatitis B virus, **HCV** - hepatitis C virus, **INR** - international normalised ratio, **Na** - sodium, **NAFLD** - non-alcoholic liver disease, **Plt** – platelets, **PS**C - primary sclerosing cholangitis, **PT** - prothrombin time. **Scoring systems: Varices:** Italian Liver Cirrhosis Project Classification (ILCP) [2] - 1: small <25% of the lumen, 2: 25-50% of the lumen, 3: large (>50%) of the lumen, **Ascites:** Child-Pugh Score for ascites - 1: none, 2: mild (or suppressed with medication), 3: moderate to severe (or refractory), **Hepatic encephalopathy (HE)** using West Haven criteria - 0: none, 1: changes in behaviour with minimal change in level of consciousness, 2: gross disorientation, drowsiness, inappropriate behaviour, 3: marked confusion, incoherent speech, sleeping but rousable to vocal stimuli, 4: comatose.

Target	Sequence	Reference
ACTB	Forward: 5’-GCACCACACCTTCTACAATGA-3’<br>Reverse: 5’-GTCATCTTCTCGCGGTTGGC-3’	
B2M	Forward: 5’-CTCGCGCTACTCTCTCTTTCT-3’<br>Reverse: 5’-CATTCTCTGCTGGATGACGTG-3’	
BMP9	Forward: 5’-GACGTCCGATAAGTCGACTACGC-3’<br>Reverse: 5’-AAGATGTGCTTCTGGAAGGGGAA-3’	[3]
BMPR2	Forward: 5’-CAAATCTGTGAGCCCAACAGTCAA-3’<br>Reverse: GAGGAAGAATAATCTGGATAAGGACCAAT-3’	
HPRT	Forward: 5’-GCTATAAATTCTTTGCTGACCTGCTG-3’<br>Reverse: 5’-AATTACTTTTATGTCCCCTGTTGACTGG-3’	
ID1	Forward: 5’-GACGGCCGAGGCGGCATG-3’<br>Reverse: 5’-GGGGAGACCCACAGAGCACG-3’	
ID2	Forward: 5’-GACCCGATGAGCCTGCTATAC-3’<br>Reverse: 5’-GGTGCTGCAGGATTTCCATCT-3’	

eTable 7: **Sequences of oligonucleotide primers used for QPCR.** All primers exhibited amplification efficiencies of 90-110%.

ID	Aetiology	Fibrosis Stage	Comorbidity	MELD-Na	UKELD	CPS	ELISA results		
							BMP9 (pg/ml)	pBMP10 (pg/ml)	sEng (pg/ml)
1	HCV/ArLD	Cirrhosis	Nil	23	60	11	20.0	500	12748
2	ArLD/A1AT	Cirrhosis	Nil	22	59	12	20.0	500	12723
3	HCV	Cirrhosis	Nil	38	70	14	20.0	500	13319
4	AIH	Mild	HTN				460.0	7366	11880
5	AIH	Mild	Nil				404.5	3859	9047
6	AIH	Mild	Osteoporosis				297.3	2352	
7	AIH/ A1AT	Cirrhosis	Hypothyroidism	7	50	5	330.0	2569	5945
8	HCV	Mild	Nil				205.6	2385	
9	AIH	Severe	Nil				198.4	767	4849
10	PBC/AIH	Cirrhosis	Nil	11	47	6	255.7	3659	9594
11	HCV/ArLD	Cirrhosis	Asthma	9	46	7	29.3	500	22492
12	HCV	Cirrhosis	Nil	6	44	6	234.8	2659	7631
13	ArLD	Cirrhosis	Nil	14	52	6	151.6	500	9687
14	HCV	Cirrhosis	Nil	7	46	5	469.5		
15	PSC	Moderate	Nil				411.0	500	5384
16	AIH	Moderate	Nil				416.4	1821	
17	ArLD	Cirrhosis	Nil	17	55	11	58.8	500	21198
18	AIH	Mild	Nil				326.0	2276	11517
19	PSC	Moderate	Systemic lupus erythematosus				425.5	2659	8109
20	PBC/AIH	Cirrhosis	Hypothyroidism	7	46	6	181.0	500	8863
21	AIH	Mild	Sjogren's				285.6		
22	PBC/AIH	Cirrhosis	Nil	9	50	6	253.3	2659	17094
23	AIH	Mild	Nil				255.1	3336	8824
24	AIH	Cirrhosis	Obesity, hypothyroidism, HTN, asthma	7	46	6	279.1	2193	11626
25	AIH	Cirrhosis	Osteoporosis	15	54	8	42.1	500	19918
26	HCV	Cirrhosis	Nil	6	46	5	211.3	2038	6666
27	AIH	Cirrhosis	Osteoporosis, adrenal insufficiency	6	45	5	221.2	3880	11497
28	AIH	Moderate	Nil				250.7	3836	12482
29	AIH	Cirrhosis	Obesity, hypothyroidism	6	43	5	222.0	2411	11394
30	AIH	Cirrhosis	Obesity	6	46	5	210.5	500	7540
31	ArLD	Cirrhosis	Nil	6	47	5	390.5	5368	17664
32	ArLD	Cirrhosis	Nil	11	51	6	226.1	1537	14033
33	HCV	Cirrhosis	Asthma	6	45	5	213.8	1146	6974
34	HCV	Cirrhosis	Nil	9	46	5	285.3	4060	5049
35	ArLD	Cirrhosis	DM type 2, HTN	7	48	5	391.8	5837	8111
36	HCV	Cirrhosis	COPD	8	47	5	177.3	500	9091
37	HCV	Cirrhosis	Myocardial infarction, HTN	8	48	5	155.7	500	6052
38	ArLD/ HCC	Cirrhotic	Nil	20	57	9	220.4	4218	9787
39	HCC	Mild	Ischaemic heart disease, atrial fibrillation, HTN				304.0	4381	4603
40	NAFLD/HCC	Cirrhosis	DM type 2, osteoporosis	21	60	10	20.0	500	15557
41	HBV/ HCC	Cirrhosis	Nil	10	47	5	239.7	2548	7147
42	ArLD/ HCC	Cirrhosis	DM type 2, HTN, hypercholesterolaemia	9	51	7	131.1	500	5598
43	HBV/ HCC	Cirrhosis	Nil	12	52	5	146.4	500	9172
44	NAFLD/HCC	Cirrhosis	Ischaemic heart disease, HTN	20	56	9	22.3	500	8715
45	ArLD/ HCC	Cirrhosis	Atrial fibrillation, HTN	16	55	9	53.8	500	13701
46	ArLD/ HCC	Cirrhosis	COPD, HTN	9	46	5	326.0	3292	7656
47	NAFLD/ArLD/HCC	Cirrhosis	COPD	7	45	6	20.0	500	5439
48	NAFLD/HCC	Severe	DM type 2				269.4	3436	3989
49	ArLD/HCC	Cirrhosis	Epilepsy	20	57	9	29.6	500	13190
50	NAFLD/ HCC	Cirrhosis	DM type 2	11	49	6	268.6	1869	3589
**51**	**PBC**	**Cirrhosis**	**Paroxysmal atrial fibrillation**	10	50	7	**20.0**	**500**	**6810**
**52**	**HCV**	**Cirrhosis**	**DM type 2, asthma**	13	51	7	**20.0**	**500**	**9743**
**53**	**HCV/ArLD/ mild HPS**	**Cirrhosis**	**DM type 2**	**20**	**56**	**10**	**20.0**	**500**	**18085**
**54**	**NAFLD/ArLD**	**Cirrhosis**	**HTN, hyperthyroidism**	23	60	11	**20.0**	**500**	**14012**
**55**	**ArLD/mod HPS**	**Cirrhosis**	**Nil**	**27**	**64**	**13**	**20.0**	**500**	
**56**	**AIH/PBC/mod HPS**	**Cirrhosis**	**Osteoporosis**	**14**	**53**	**10**	**74.1**	**500**	**15399**
**57**	**NASH**	**Cirrhosis**	**DM type 2, HTN, hypercholesterolaemia, hypothyroidism**	19	55	9	**20.0**	**500**	**10487**
**58**	**ArLD**	**Cirrhosis**	**Nil**	12	**52**	8	**20.0**	**500**	**12623**
**59**	**HCV/ArLD**	**Cirrhosis**	**DM type 2**	10	47	9	**125.4**	**500**	
**60**	**HBV/NAFLD**	**Cirrhosis**	**Nil**	15	54	8	**101.9**	**500**	
**61**	**ArLD/mod HPS**	**Cirrhosis**	**Nil**	**15**	**54**	**11**	**20.0**	**500**	**17131**
**62**	**ArLD/mild HPS**	**Cirrhosis**	**Nil**	**19**	**57**	**10**	**20.0**	**500**	**12815**
**63**	**HCV/PoPH**	**Cirrhosis**	**Nil**	**19**	**57**	**8**	**20.0**	**500**	**10565**
**64**	**Sarcoid**	**Cirrhosis**	**DM type 2, hypothyroidism**	**10**	**51**	**8**	**45.5**	**500**	**6037**
**65**	**PSC**	**Cirrhosis**	**Nil**	**10**	**48**	**9**	**20.0**		

66	NAFLD/mod HPS	Cirrhosis	Nil	19	57	13	83.1	500	12887
67	HCV	Cirrhosis	HTN, hypothyroidism, asthma, osteoporosis	7	48	6	329.4	1333	5987
68	NAFLD	Cirrhosis	DM type 2, obesity	14	53	6	368.5	500	11074
69	ArLD/severe HPS	Cirrhosis	DM type 2	16	54	7	90.5	500	
70	ArLD/PoPH	Cirrhosis	Nil	18	56	7	20.0		
71	ArLD/mod HPS	Cirrhosis	DM type 2, hypercholesterolaemia	23	60	10	20.0	500	18860
72	ArLD	Cirrhosis	DM type 2, atrial fibrillation	11	50	8	77.2	500	6504
73	HCV/mod HPS	Cirrhosis	Nil	17	56	11	20.0		
74	ArLD	Cirrhosis	DM Type 2	19	57	10	20.0	500	
75	ArLD/mod HPS	Cirrhosis	Haemorrhagic stroke, HTN	18	54	11	40.4	500	9038
76	Sarcoid/v severe HPS	Cirrhosis	DM type 2, obesity	13	51	6	20.0	500	5498
77	NAFLD	Cirrhosis	DM Type 2, HTN	15	53	9	20.0	500	12362
78	ArLD	Cirrhosis	Nil	14	54	9	20.0	500	
79	NAFLD	Cirrhosis	Obesity, obstructive sleep apnoea	15	55	13	20.0		
80	ArLD/mod HPS	Cirrhosis	Subdural haematoma	26	61	12	20.0	500	11896
81	ArLD	Cirrhosis	Nil	30	67	13	20.0	500	10184
82	A1AT/v severe HPS	Cirrhosis	HTN	13	52	6	20.0	500	11359
83	HCV/mild HPS	Cirrhosis	Nil	11	50	9	69.8	500	7594
C1							366.3	500	10323
C2							264.2	500	10772
C3							335.6	3788	14398
C4							215.9	503	7937
C5							248.4	500	18679
C6							370.1	5273	9689
C7							329.4	500	9033
C8							266.0	2036	5478
C9							347.4	6267	6829
C10							218.3		
C11							197.5	2018	7847
C12							255.4		
C13							193.8	2034	7141
C14							348.3	1433	8757
C15							302.5	3093	4420
C16							289.5	3088	6140
C17							354.0	4871	6318
C18							343.3	4678	6223
C19							337.5	7917	4498
C20							268.6	3135	4874
C21							194.4	1754	14816

eTable 8: Comorbidities, liver disease severity scores and measured values for BMP9, pBMP10 and sEng for individual cirrhotic and fibrotic patients and non-disease controls assessed in this study.
The demographics for all patients (n=83) assessed for fibrosis/cirrhosis are detailed. Of these, patients 51-83 were assessed for cardiopulmonary parameters by right heart catheterisation, portal venous pressure measurements and bubble echocardiogram. Individuals C1-21 are the controls. Those patients highlighted in blue have hepatopulmonary syndrome (HPS, n=14) with severity indicated for individual patients (mod = moderate, v = very) and those in red have PoPH (n=2). **Aetiology Abbreviations: A1AT** - alpha 1 anti-trypsin, **AIH** - autoimmune hepatitis, **ALB** – albumin, **ArLD** - alcohol related liver disease, **Asc** – ascites, **Cr** – creatine, **HBV** - hepatitis B virus, **HCC** - hepatocellular carcinoma, **HCV** - hepatitis C virus, **INR** - international normalised ratio, **Na** - sodium, **NAFLD** - non-alcoholic liver disease, **PBC** - primary biliary cholangitis, **Plt** – platelets, **PSC** - primary sclerosing cholangitis, **PT** - prothrombin time. **Comorbidity Abbreviations: COPD** – chronic obstructive pulmonary disease, **DM type 2** – Diabetes Mellitus type 2**, HTN** – systemic hypertension. **Scoring systems: MELD-Na -** Model for End-Stage Liver Disease[4], **UKELD** – United Kingdom Model for End-Stage Liver Disease[5], **CPS** - Child-Pugh Score[6].

		Non-HPS Liver Disease Severity Scores	HPS Liver Disease Severity Scores	*P* value
MELD-Na[4]	BMP9 samples	13.00 [10.00;15.00], n=15	17.50 [13.75;20.75], n=14	0.0091
	pBMP10 samples	13.50 [10.00;16.00], n=14	18.00 [13.50;21.50], n=13	0.0191
	sEng samples	12.50 [10.00;16.00], n=10	18.00 [13.00;20.00], n=11	0.0364
UKELD[5]	BMP9 samples	49.00 [46.00;52.00], n=15	53.50 [51.00;57.00], n=14	0.0047
	pBMP10 samples	49.50 [46.75;52.25], n=14	54.00 [51.00;57.00], n=13	0.0107
	sEng samples	48.00 [46.75;52.25], n=10	52.00 [51.00;57.00], n=10	0.0271
Child-Pugh Score[6]	BMP9 samples	8.00 [7.00;9.00], n=15	10.00 [8.50;11.25], n=14	0.0077
	pBMP10 samples	8.00 [7.00;9.00], n=14	10.00 [8.00;11.50], n=13	0.0150
	sEng samples	8.00 [6.75;8.25], n=10	10.00 [9.00;11.00], n=10	0.0080

eTable 9: Liver disease severity scores for cirrhotic patients classified as non-HPS and HPS.
Liver disease severity scores were calculated for each patient. Sample numbers were lower for pBMP10 and sEng due to limited sample volume in some samples. Data are presented as “Median [Interquartile range], number”. Statistical comparison of non-HPS and HPS was by Mann-Whitney test. Exact P values were calculated using GraphPad Prism 8.2.1. **MELD-Na** - Model for End-Stage Liver Disease, **UKELD** - United Kingdom Model for End-Stage Liver Disease, **CPS** - Child-Pugh Score.

SUPPLEMENTAL FIGURES

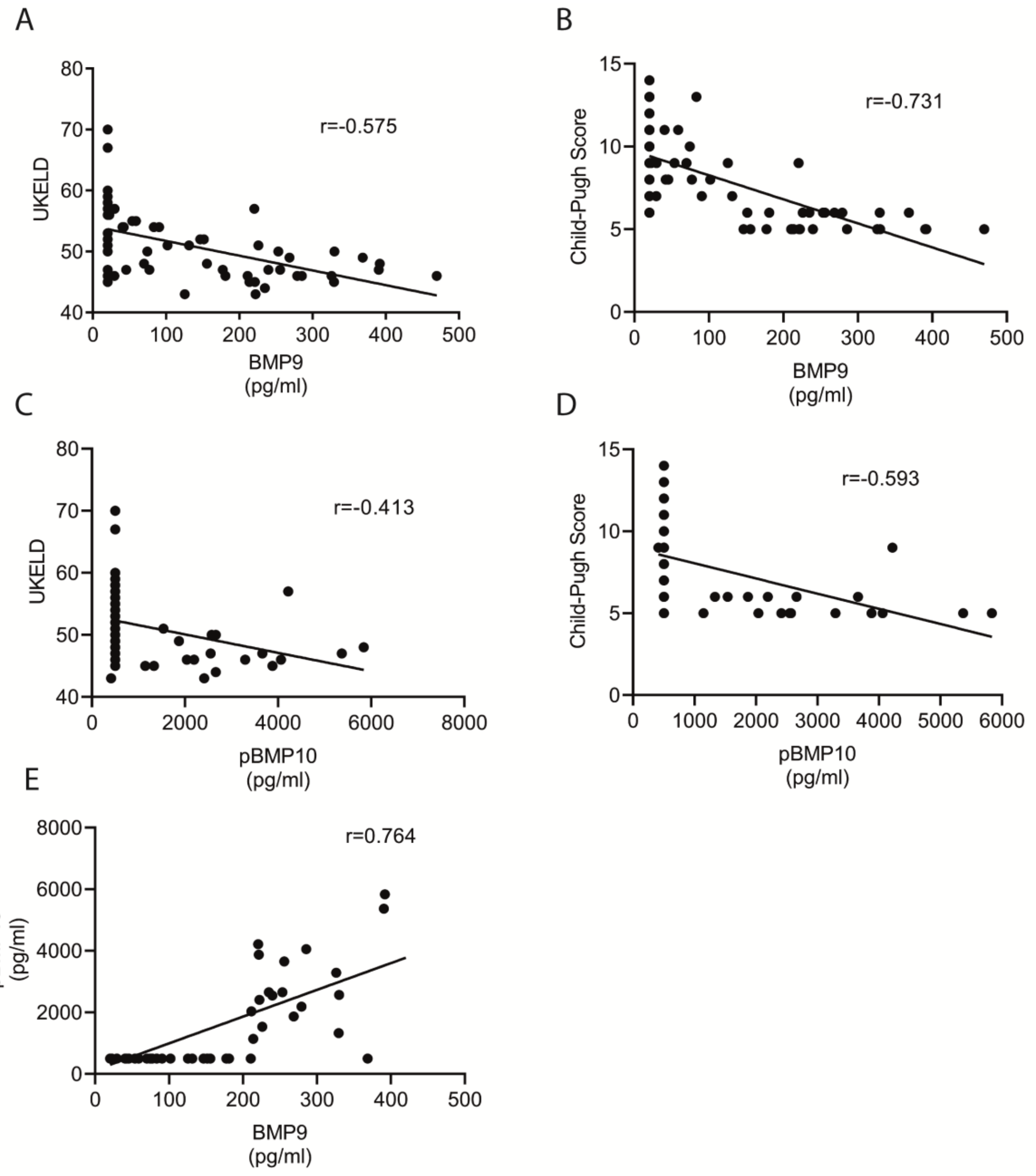

eFigure 1: BMP9 and pBMP10 levels correlate to the severity of liver disease and to each other.

(A,B) Plasma BMP9 levels measured in cirrhotics (n=69) correlated to (A) UKELD and (B) CPS. (C,D) Plasma pBMP10 levels measured in cirrhotics (n=64) correlated to (C) UKELD (*P*=0.0007) and (D) CPS. (E) Plasma BMP9 and pBMP10 levels correlate with each other (n=64). Spearman correlation *P*<0.0001 for all graphs except where stated otherwise.

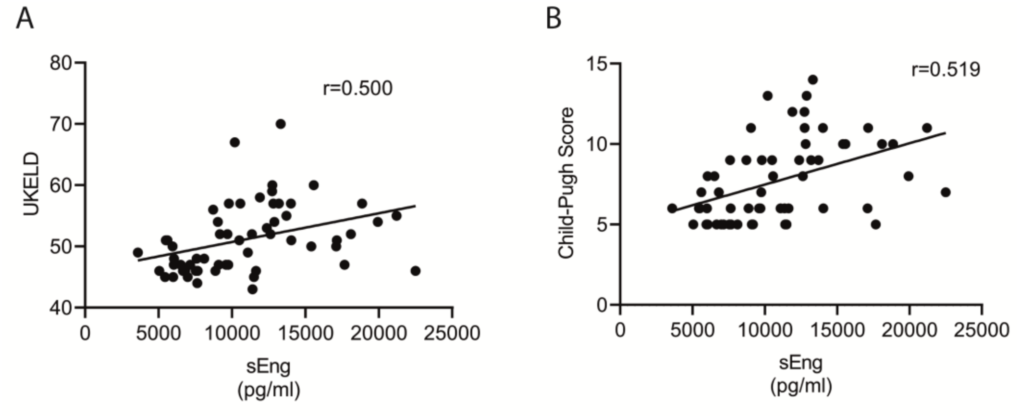

eFigure 2: sEng levels correlate to the severity of liver disease.

(A,B) Plasma sEng levels measured in cirrhotics (n=58) correlated to (A) UKELD and (B) CPS. Spearman correlation $P<0.0001$ for all graphs.

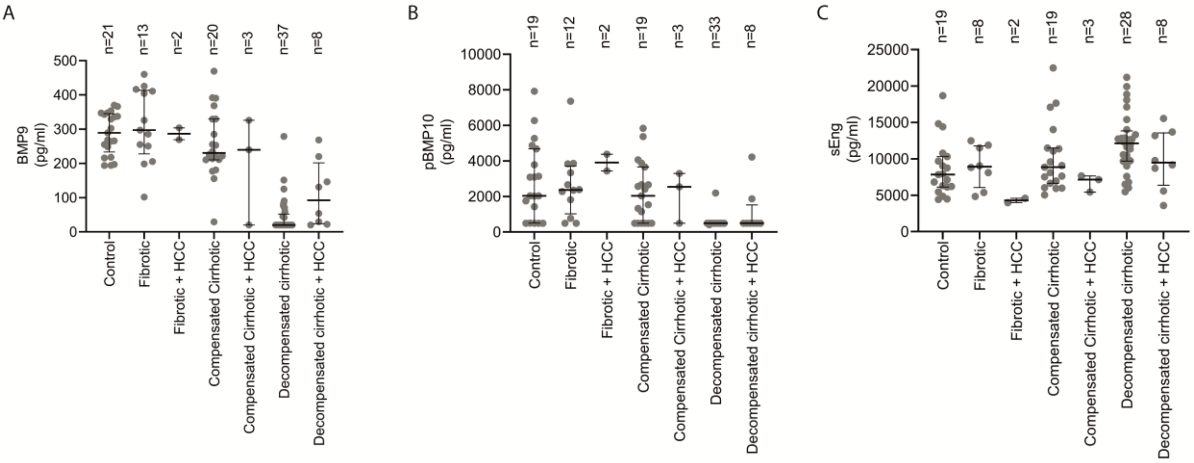

eFigure 3. Circulating BMP9, pBMP10 and sEng do not significantly differ between patients with or without hepatocellular carcinoma.

Plasma samples from controls and patients with cirrhosis were assayed by ELISA for (A) BMP9, (B) pBMP10 or (C) sEng. The data for fibrotics, compensated cirrhotics and decompensated cirrhotics were divided into those patients with HCC and those without HCC. Error bars show median and interquartile range. Kruskal Wallis test for comparison of each group without or with HCC were not significant. Numbers are cited above each dataset. HCC = hepatocellular carcinoma

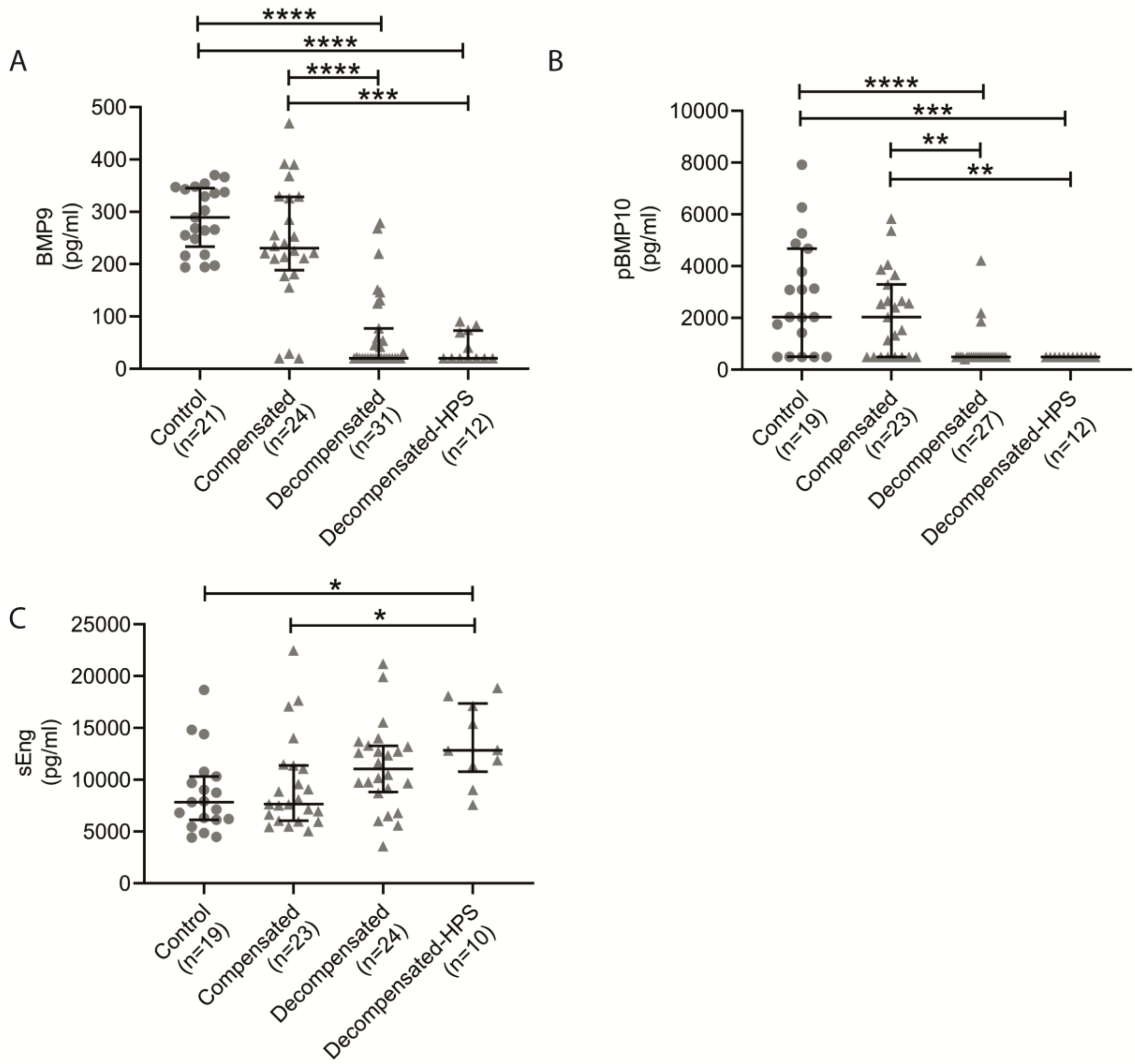

eFigure 4. Circulating BMP9 and pBMP10 are reduced to similar extends in decompensated cirrhotics with or without HPS and sEng levels are elevated in both groups.

Plasma samples from controls and patients with cirrhosis were assayed by ELISA for (A) BMP9, (B) pBMP10 or (C) sEng and the data for decompensated cirrhotics divided into those without HPS and those with HPS. The data for the controls and compensated cirrhotics are included for reference. Error bars show median and interquartile range. Kruskal Wallis test: *P<0.05, **P<0.01, ***P<0.001, ****P<0.0001, Error bars show median and interquartile range. HPS = hepatopulmonary syndrome.

A

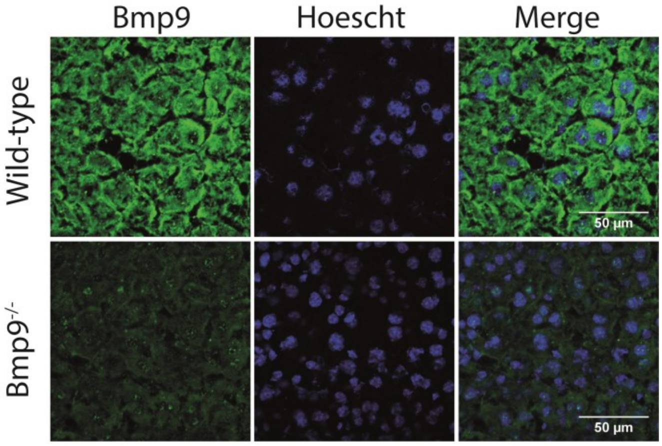

B

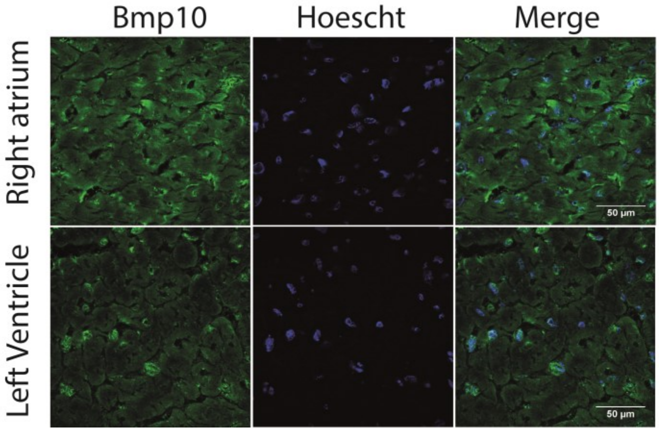

eFigure 5. Validation of the BMP9 and BMP10 antibodies used for immunohistochemistry.

(A) Liver sections from Bmp9-/- knock-out mice (n=3) and wild type littermates (n=3) were stained using the BMP9 antibody. No BMP9 staining was observed seen in the *Bmp9* knock-out mouse.(B) Sections of human right atrium and left atrium were stained using a BMP10 antibody. Consistent with previous reports of expression, BMP10 staining was more intense in the right atrium compared to the left atrium.

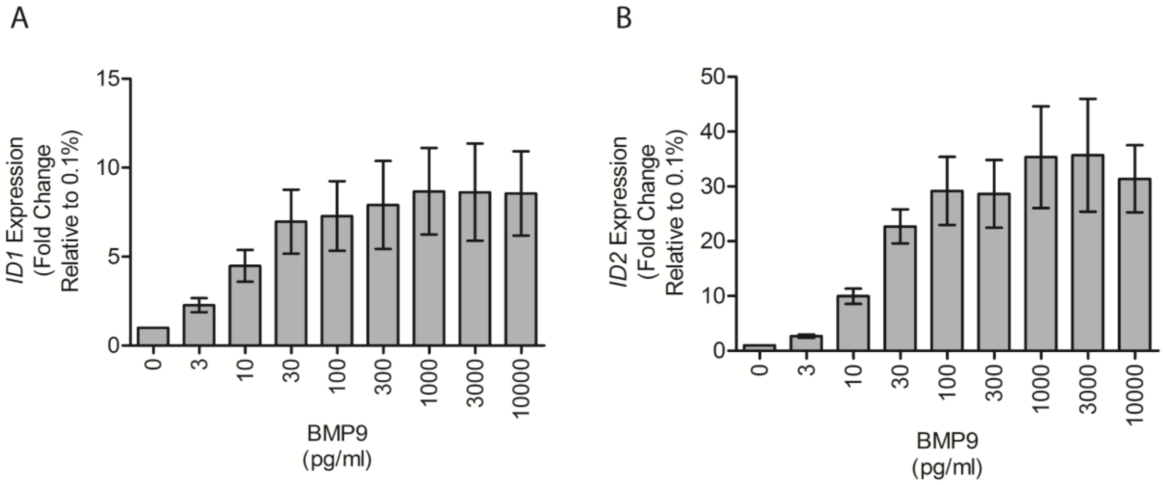

eFigure 6: BMP9 stimulates *ID* gene transcription to a maximum at 100pg/ml in HAECs.

(A, B) HAECs were serum-depleted overnight followed by addition of 3-10000 pg/ml BMP9. After 1h, cells were lysed, RNA extracted and cDNA analysed for the expression of (A) *ID1* or (B) *ID2*. (n=3 experiments).

REFERENCES

1. Sen S, Alexander GJM. Pulmonary complications in liver disease. *Brit J Hosp Med* 2009; **70**(12): 685-91.
2. Philips CA, Sahney A. Oesophageal and gastric varices: historical aspects, classification and grading: everything in one place. *Gastroenterol Rep* 2016; **4**(3): 186-95.
3. Bidart M, Ricard N, Levet S, et al. BMP9 is produced by hepatocytes and circulates mainly in an active mature form complexed to its prodomain. *Cell MolLife Sci* 2012; **69**(2): 313-24.
4. Biggins SW, Kim WR, Terrault NA, et al. Evidence-based incorporation of serum sodium concentration into MELD. *Gastroenterology* 2006; **130**(6): 1652-60.
5. Barber K, Madden S, Allen J, et al. Elective Liver Transplant List Mortality: Development of a United Kingdom End-Stage Liver Disease Score. *Transplantation* 2011; **92**(4): 469-76.
6. Cholongitas E, Papatheodoridis GV, Vangeli M, Terreni N, Patch D, Burroughs AK. Systematic review: the model for end-stage liver disease - should it replace Child-Pugh's classification for assessing prognosis in cirrhosis? *Aliment Pharm Therap* 2005; **22**(11-12): 1079-89.
